# A Transcriptomic Model of Postnatal Cardiac Effects of Prenatal Maternal Cortisol Excess in Sheep

**DOI:** 10.3389/fphys.2019.00816

**Published:** 2019-07-03

**Authors:** Andrew Antolic, Elaine M. Richards, Charles E. Wood, Maureen Keller-Wood

**Affiliations:** ^1^Department of Pharmacodynamics, University of Florida, Gainesville, FL, United States; ^2^Department of Physiology and Functional Genomics, University of Florida, Gainesville, FL, United States

**Keywords:** cortisol, pregnancy, maternal stress, cardiac metabolism, adipose, lipid

## Abstract

*In utero* treatment with glucocorticoids have been suggested to reprogram postnatal cardiovascular function and stress responsiveness. However, little is known about the effects of prenatal exposure to the natural corticosteroid, cortisol, on postnatal cardiovascular system or metabolism. We have demonstrated an increased incidence of stillbirth in sheep pregnancies in which there is mild maternal hypercortisolemia caused by infusion of 1 mg/kg/d cortisol. In order to model corticosteroid effects in the neonate, we created a second model in which cortisol was infused for 12 h per day for a daily infusion of 0.5 mg/kg/d. In this model we had previously found that neonatal plasma glucose was increased and plasma insulin was decreased compared to those in the control group, and that neonatal ponderal index and kidney weight were reduced and left ventricular wall thickness was increased in the 2 week old lamb. In this study, we have used transcriptomic modeling to better understand the programming effect of this maternal hypercortisolemia in these hearts. This is a time when both terminal differentiation and a shift in the metabolism of the heart from carbohydrates to lipid oxidation are thought to be complete. The transcriptomic model indicates suppression of genes in pathways for fatty acid and ketone production and upregulation of genes in pathways for angiogenesis in the epicardial adipose fat (EAT). The transcriptomic model indicates that RNA related pathways are overrepresented by downregulated genes, but ubiquitin-mediated proteolysis and protein targeting to the mitochondria are overrepresented by upregulated genes in the intraventricular septum (IVS) and left ventricle (LV). In IVS the AMPK pathway and adipocytokine signaling pathways were also modeled based on overrepresentation by downregulated genes. Peroxisomal activity is modeled as increased in EAT, but decreased in LV and IVS. Our results suggest that pathways for lipids as well as cell proliferation and cardiac remodeling have altered activity postnatally after the *in utero* cortisol exposure. Together, this model is consistent with the observed increase in cardiac wall thickness at necropsy and altered glucose metabolism observed *in vivo*, and predicts that *in utero* exposure to excess maternal cortisol will cause postnatal cardiac hypertrophy and altered responses to oxidative stress.

## Introduction

Cortisol plays an important role in the maturation of many fetal organs, and in primates and many other mammalian species, including the sheep, is essential for the normal maturation of organs such as the lung which are essential for the transition to ex utero life (Liggins, 1994). The heart is one of the organs in which cortisol appears to play a vital role in maturation (Rog-Zielinska et al., 2013). However, excess glucocorticoids or repeated exogenous glucocorticoid treatments, have been found to reduce birth weight (Newnham and Moss, 2001; Davis et al., 2009), increase postnatal blood pressure (Dodic et al., 1998, 2001, 2002a,b), and alter postnatal glucose metabolism (Moss et al., 2001; Sloboda et al., 2002; Antolic et al., 2015).

Late gestation is a critical period for maturation of cardiomyocytes. During this period cardiomyocytes transition from primarily mononucleated and proliferative cells to binucleated, terminally differentiated cardiomyocytes (Jonker et al., 2007b). Circulating factors that stimulate proliferation include angiotensin II, cortisol, and insulin-like growth factor-1, while atrial natriuretic peptide (ANP) and tri-iodo-L-thyronine (T3) suppress proliferation (Sundgren et al., 2003a, b; Giraud et al., 2006; Chattergoon et al., 2007; OTierney et al., 2010b). Hemodynamic forces also influence cardiomyocyte proliferation; proliferation is stimulated by increased arterial pressure and suppressed with reduced systolic load (Jonker et al., 2007a; OTierney et al., 2010a). After birth there are substantial changes that occur in cardiac metabolism as the nutrient supply dramatically changes. Throughout gestation and immediately after birth the heart relies almost entirely on glycolysis and lactate oxidation, with minimal contribution from β-oxidation of fatty acids due to their limited supply (Werner and Sicard, 1987; Lopaschuk et al., 1991; Bartelds et al., 1998, 2000). However, in the newborn period, there is a gradual shift in the reliance on glucose and lactate for carbon sources to β-oxidation of fatty acids supplied in the mother’s milk (Werner and Sicard, 1987; Ascuitto et al., 1989; Lopaschuk and Spafford, 1990; Lopaschuk et al., 1991). Thus, changes in myocyte maturation, but also afterload and metabolism all occur in the immediate neonatal period. In our previous studies in pregnant ewes, we have shown that increased levels of maternal cortisol in late gestation, similar to levels expected in a chronically stressed animal, alter the trajectory of cardiac gene expression and indicated that metabolic pathways were overrepresented in the differentially expressed genes in fetuses near term (Richards et al., 2014). In our sheep model, a chronic doubling of maternal cortisol over the last month of fetal life was associated with a dramatic increase in stillbirth in the immediate perinatal period (Keller-Wood et al., 2014), and with significantly lower heart rate in the last hour before birth (Antolic et al., 2017). The newborns of cortisol-treated ewes also have changes in the cardiac transcriptome and in the cardiac and serum metabolomes (Antolic et al., 2019; Walejko et al., 2019); these data have suggested that metabolism of lipids and the TCA cycle are altered in the fetuses of ewes with higher than normal cortisol concentrations. Transcriptomic modeling of pathways in the heart altered after maternal cortisol treatment also indicated increased expression of genes associated with the TGFβ pathway, consistent with the observed increase in collagen deposition and wall thickness of the left ventricular free wall in the newborns after maternal cortisol treatment (Antolic et al., 2019).

To determine postnatal effects of increased *in utero* cortisol exposure, we used a model in which ewes were treated with cortisol for 12 h per day, starting in the evening and continuing into the next morning. This model is less severe than the model in which ewes are continuously infused with cortisol, but the design mimics the pattern of cortisol in Cushing’s syndrome in which evening cortisol levels fail to fall (Laudat et al., 1988; Raff et al., 1998; Scharnholz et al., 2010). Although sheep do not have a circadian rhythm in cortisol (Bell et al., 1991), in most animal housing situations we expect a morning rise in cortisol during feeding and husbandry, with lower levels in the evening and overnight hours. We found that in newborns of this experimental paradigm, there was an increase in the relative thickness of the LV wall at 2 weeks of age relative to that of 2 week old lambs of control ewes. There is an increase in the glucose to insulin ratio in the offspring of the cortisol-treated ewes, which may also modify cardiac metabolism. We used a transcriptomic approach to model the effects of the *in utero* exposure to assess which pathways in the postnatal heart are altered by the prior exposure. Specifically, we were interested in whether pathways related to carbohydrate or fatty acid oxidation, or to structural changes in the heart were significantly over-represented. Because our previous studies with more chronic exposure to high maternal cortisol (continuous infusion at 1 mg/kg/d to the ewe) produced effects on cardiac transcriptomics and metabolomics suggesting altered energy utilization, we included samples from the epicardial fat depot to assess the changes in gene expression in that tissue, and model the pathways altered in the epicardial adipose with those in the nearby cardiac tissue. These studies were designed to inform future studies in offspring from this model of maternal stress, by using gene expression patterns to provide an untargeted and unbiased insight into changes in the offspring heart.

## Materials and Methods

### Experimental Design

All animal use was approved by the Institutional Animal Care and Use Committee at the University of Florida. Black-faced ewes with singleton (13) or twin pregnancies (2) were studied in late gestation. During study, ewes were housed indoors in a facility with temperature and humidity-controlled rooms, and with controlled light cycle (lights on 0700 to 1900 h), and animals were housed in pens that allowed them free movement throughout the pregnancy and postpartum period. Ewes were of similar body condition and were fed a diet of pelleted feed at a weight within NRC standards for the ewe’s body weight, fetal number and gestational age, with supplementation with alfalfa hay from 140 day onward. Ewes were prepared with chronic indwelling femoral arterial and venous catheters at approximately day 115 of pregnancy under isoflurane anesthesia as previously described (Antolic et al., 2015). Ewes were randomly assigned at surgery to one of two groups: control with no infusion (Control; *n* = 7 ewes), or cortisol- infused (CORT, *n* = 10 ewes; Cortisol (Solu-Cortef, Pfizer, Inc.). Cortisol was infused intravenously using a syringe pump into the maternal femoral arterial catheter at a total dose of 0.5 mg/kg/d; the pumps were controlled with a timer (Chronotrol, ChronTrol Corporation, San Diego, CA, United States) to restrict the infusions to 2100 to 0900 h. All infusions were delivered to the ewes in their pens using a tether system for the infusion; this allows the ewes to freely move about their pens throughout the period of the study. The cortisol infusion was discontinued after delivery of the lamb(s). Lambs were housed with their ewes in the same pen; on day 3 after birth the pen was opened to an adjacent pen to allow the ewe and her lamb additional space.

The *in vivo* data and morphometrics at 2 weeks of age in the control and cortisol-treated ewes and their lambs has been previously reported (Antolic et al., 2015). The average morning cortisol concentration during the infusion was 22.0 ± 4.0 ng/ml in CORT ewes and 9.5 ± 2.6 ng/ml in the Control ewes. Two lambs in the CORT group were stillborn. One set of twins were born in each group of ewes. Seven control ewes delivered four male and four female lambs at 144–149 days gestation; eight CORT ewes delivered four male and five female lambs at 142–146 days. Ewes were sampled before birth, and lambs were sampled after birth using a jugular line which was placed on the day of birth, as previously described. As previously reported, there was no effect of this maternal cortisol infusion on the lambs’ body temperature, or body weight at birth or on the following days. Although ponderal index was reduced overall in the lambs in the CORT group, this effect was only significant in the male lambs. Although morning maternal cortisol concentrations were increased in the ewes prior to birth, plasma cortisol concentrations were not different postpartum in the ewes or between the two groups of lambs. There was an overall effect of CORT to increase plasma glucose and decrease plasma insulin concentrations in the postnatal lambs, and therefore average glucose to insulin ratio was increased in the CORT lambs, as previously reported (Antolic et al., 2015).

On postnatal day 14–15, the lambs were killed via intravenously administered Euthasol solution (Euthasol; Fort Worth, TX, United States). Organ weights were measured at necropsy. As previously reported (Antolic et al., 2015) cardiac weight was not significantly altered by CORT, but the ratio of left ventricular free wall thickness relative to tibial length was increased (control: 0.445 ± 0.017 mm/cm vs. CORT: 0.496 ± 0.019 mm/cm). There were no significant differences in relative septal wall thickness (control: 0.512 ± 0.030 mm/cm vs. CORT: 0.530 ± 0.021 mm/cm) or relative right ventricular wall thickness (control: 0.235 ± 0.018 mm/cm vs. CORT: 0.253 ± 0.010 mm/cm).

### Tissue Collection for Transcriptomic Analysis

At necropsy, the hearts were quickly removed and dissected using sterile instruments and gloves (*n* = 8 per group). Tissue samples taken from the interventricular septum (IVS), left ventricular free wall (LV) and the epicardial adipose depot (EAT) were snap frozen and stored at −80°C until analysis.

Messenger RNA from homogenized samples of lamb left ventricular free wall, septa, and epicardial adipose were extracted using Trizol and then purified with on-column DNase digestion (Qiagen RNeasy Plus kits; Qiagen Sciences, Germantown, MD, United States). RNA integrity numbers (RIN) for the RNA samples were between 7.3 and 8.3 for LV, 7.7–8.6 for IVS (*n* = 8 control and 8 CORT for each section of the heart) and 7.1–7.7 for EAT samples (*n* = 7 control, *n* = 9 CORT). RNA (500 ng) was labeled with Cy5 using the Agilent QuickAmp Labeling kit; hybridization was performed using previously described methods (Richards et al., 2014). We used the Agilent-019921 Sheep Gene Expression Microarray 8 × 15 k, G4813A (GPL14112) platform. Our group has described the annotation for this array platform in previous publications (Richards et al., 2014; Chang et al., 2018). The raw normalized array data for this experiment have been deposited in the National Center for Biotechnology Information’s Gene Expression Omnibus (GEO) and are accessible through GEO Series accession number GSE131537 for the cardiac samples, and GEO Series accession number GSE119254 for the epicardial fat samples.

### Microarray Data Analysis

Raw microarray data was processed using the Limma package in R software (R Development Core Team, 2008), to perform background correction and data normalization using the quantile method. Probes with low expression and microarray control probes were excluded as well as those probes that were less than 10% brightness of the negative controls. The remaining probes were then retained for further statistical analysis.

### Statistical Analysis

In order to identify differentially expressed genes in the LV and IVS the Limma package was utilized to analyze the processed microarray data using a moderated *t*-test that employs an empirical Bayes method (*P* < 0.05). The effect of cortisol was analyzed by comparing the gene expression in tissues collected from CORT lambs compared to Control lambs.

### Network and Enrichment Analysis

Webgestalt was used to identify overrepresented biological processes, molecular functions, and cellular components associated with the differentially regulated genes. *P* = 0.05 was used as the criterion for statistical significance after Benjamini-Hochberg multiple test correction of the *p*-value, with a minimum of two genes per category (Zhang et al., 2005; Wang et al., 2013). This approach included all genes that were significant at *p* < 0.05 without correction for multiple comparisons, and corrects for the false discovery in the pathway analyses by using the Benjamini-Hochberg correction for false discovery due to multiple testing. GeneMANIA, BiNGO, and ClueGO plugins of Cytoscape, were used to perform network inference of the differentially regulated genes, gene ontology analysis, and generate enriched gene-gene interaction networks from GO and KEGG databases (Shannon et al., 2003; Bindea et al., 2009; Montojo et al., 2010; Mlecnik et al., 2018). The results of these analyses were considered significantly different between the CORT and Control tissues using *p* < 0.05 corrected for false discovery rate.

## Results

### Genes Altered by Maternal Cortisol Infusion in the Left Ventricular Free Wall of the Postnatal Lamb

We found 115 genes were upregulated and 145 genes were downregulated in the LV of the 14-day-old lambs of cortisol-treated ewes as compared to lambs of control ewes (Supplementary Table 1). Webgestalt analysis found that the only major molecular function upregulated in the left ventricle by overnight maternal cortisol was peptidyl-prolyl *cis*-*trans* isomerase activity (4 genes; adj. *P* = 7.30E-03). Overrepresented cellular components represented by upregulated genes in the lamb LV after maternal cortisol infusion included cytoplasm (26 genes), mitochondria (22 genes), peroxisome (4 genes), and nucleus (51 genes). More specific components include mitochondrial outer membrane translocase complex (2 genes; adj. *P* = 6.60E-03), intrinsic to mitochondrial outer membrane (2 genes; adj. *P* = 1.56E-02), integral to peroxisomal membrane (2 genes; adj. *P* = 1.47E-02), and nuclear lumen (27 genes; adj. *P* = 1.68E-02). Gene Ontology analysis identified negative regulation of transforming growth factor beta receptor signaling pathway (4 genes; adj. *P* = 4.78E-02, including NR3C1 encoding GR), protein targeting mitochondrion (4 genes; adj. *P* = 4.78E-02, including TOMM40, TIMM8A, TOMM5, and MFN2), and response to arsenic-containing substance (3 genes; adj. *P* = 4.78E-02), ribonucleotide and ribonucleoside biosynthetic process (5 genes; adj. *P* = 4.78E-02), cellular protein modification process (31 genes; adj. *P* = 4.78E-02), fatty acid oxidation (2 genes; adj. *P* = 4.78E-02) as pathways overrepresented by upregulated genes in the LV in CORT lambs compared to Control lambs. KEGG pathway analysis (Table 1) identified pathways associated with the upregulated genes in the postnatal lamb LV of the CORT lambs compared to Control lambs. The identified pathways were ubiquitin mediated proteolysis, metabolic pathways, peroxisome, drug metabolism-other enzymes, insulin signaling pathway, RIG-I-Like receptor signaling pathway, and phosphatidylinositol signaling system.

**TABLE 1:** KEGG pathways significantly overrepresented in up and down-regulated genes in LV of 2-week-old lambs from cortisol-treated ewes (0.5 mg/kg/day) compared to lambs of control ewes(*n* = 8 lambs per group; 4 males and 4 females).

**Up-regulated pathway names**	**#Genes**	***P*-value**	**Genes**
Ubiquitin mediated proteolysis	6	2.90E-05	RCHY1, KLHL9, TCEB2, ANAPC13, BIRC6, UBA2.
Peroxisome	3	0.011	PEX13, SLC27A, AGPS
Metabolic pathways	8	0.0356	HPRT1, ACACB, AGPS, UCKL1, MMAB, PHOSPHO2, ALG1, NME2
Drug metabolism	2	0.0356	HPRT1, UCKL1
Insulin signaling pathway	3	0.0356	CALM2, ACACB, SREBF1
RIG-I-like receptor signaling pathway	2	0.0356	DDX3X, PIN1
Phosphatidylinositol signaling pathway	2	0.0356	CALM2, PIP4K2A

**Down-regulated pathway names**			

Neuroactive ligand-receptor interaction	5	0.007	THRB, PTGER3, TSPO, GHR, ADRB1
RNA transport	4	0.007	EIF4G1, EELAC1, EIF3A, POP7
Ribosome biogenesis in eukaryotes	3	0.007	DKC1, IMP4, POP7
Circadian rhythm – mammal	2	0.007	CLOCK, CSNK1E
Phagosome	4	0.007	TUBB2B, CYBA, TUR6, TUBB3
RNA degradation	3	0.007	EXOSC2, CNOT10, DCP2
Gap junction	3	0.0081	TUBB2B, ADRB1, TUBB3
Base excision repair	2	0.0119	MPG, NTHL1
Pathogenic *Escherichia coli* infection	2	0.0238	TUBB2B, TUBB3
Amyotrophic lateral sclerosis (ALS)	2	0.0238	DERL1, BAX
Hedgehog signaling pathway	2	0.0238	CSNK1G2, CSNK1E
Calcium signaling pathway	3	0.0302	PTGER3, PLCD1, ADRB1
Metabolic pathways	8	0.0412	GART, PIGT,PLCD1, ST6GAL1, PPAP2C, DHCR7, CYP27A1, CRYL1
Salivary secretion	2	0.0441	ATP1B3, ADRB1
Ribosome	2	0.0441	RPS8, RPS2

Overrepresented cellular components reflected by downregulated genes in the LV of CORT lambs compared to Control lambs were macromolecular complex (46 genes), cytosol (29 genes), nucleoplasm (23 genes) and nucleolus (24 genes). The only overrepresented biological processes related to the downregulated genes in the CORT lambs compared to Control lambs were macromolecular catabolic processes (70 genes; *P* = 4.53E-02) and RNA processing (15 genes, *p* = 4.53E-02). There were no molecular functions associated with downregulated pathways in the LV of CORT lambs. Pathways identified for the downregulated genes in CORT lambs compared to Control lambs were neuro ligand-receptor interaction, RNA transport, ribosome biogenesis in eukaryotes, circadian rhythm-mammal, phagosome, RNA degradation, gap junction, base excision repair, pathogenic E. coli infection, amyotrophic lateral sclerosis, hedgehog signaling pathway, calcium signaling pathway, metabolic pathways, salivary secretion, and ribosome (Table 1).

**FIGURE 1:**
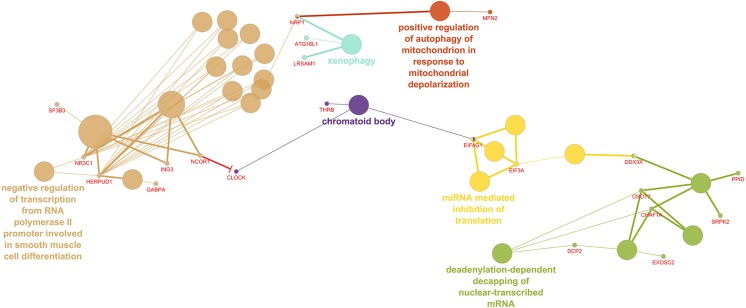
Enriched ClueGO gene network from the left ventricular free wall. Similar gene ontology or KEGG pathway terms are clustered by color and the representative term for those clusters are presented. Gene names shown in red; Red lines with bars represent interactions involved in gene repression.

The 260 differentially regulated genes in the LV were entered into ClueGO, which recognized 255 genes as having functional annotations. The 255 genes were significantly associated with 40 gene ontology terms or KEGG pathways and further organized into 18 groups based on the similarity of their associated genes, which were used to create an enriched network based on gene-gene interactions. The most significant term from each group was used as the representative term (Figure 1). The group with the most terms associated to it was the negative regulation of transcription from RNA polymerase II promoter involved in smooth muscle cell differentiation (2 genes, adj. *P* = 3.88E-04).

### Genes Altered by Maternal Cortisol Treatment in the Interventricular Septum of the Postnatal Lamb

We found 103 genes were upregulated and 153 were downregulated in the IVS of 14-day-old lambs of cortisol infused ewes compared with 14-day-old lambs of untreated ewes (Supplementary Table 2). Major cellular components associated with the genes upregulated in the CORT lambs compared to Control lambs were cytosol, mitochondria and ribosome. The only major molecular function modeled as upregulated by the maternal cortisol treatment was structural constituent of ribosome (6 genes; adj. *P* = 1.07E-02; this included expression of genes encoding 4 mitochondrial ribosomal proteins, L2, L33, S11, and S33). Gene Ontology analysis identified cellular protein transport (16 genes *p* = 1.62E-20) as a significantly altered biological process associated with upregulated genes in the CORT lambs compared to Control lambs, and more specifically identified protein targeting to the mitochondria (4 genes, *P* = 2.56E-20; including TIMM8A, TIMM8B, TOMM5, and MTX1) as well as chaperone-mediated protein transport (2 genes; adj. *P* = 4.35E-02), and regulation of protein ubiquitination (7 genes; adj. *P* = 1.62E-02) as upregulated in CORT lambs. KEGG pathway analysis (Table 2) indicated that this maternal cortisol infusion upregulated pathways in the postnatal lamb IVS involving protein processing in the endoplasmic reticulum, ubiquitin-mediated proteolysis, p53 signaling pathway, the ribosome, and mRNA surveillance pathway.

**TABLE 2:** KEGG pathways significantly overrepresented in up anddown-regulated genes in IVS of 2-week-old lambs from cortisol-treated (0.5 mg/kg/day) ewes compared to lambs of control ewes(*n* = 8 lambs per group; 4 males and 4 females).

**Up-regulated pathway names**	**#Genes**	***P*-value**	**Genes**
Protein processing in endoplasmic reticulum	4	0.006	SEC13, ERP29, SEC24C, HERPUD1
Ubiquitin mediated proteolysis	3	0.0195	RCHY1, TCEB1, ANAPC11
p53 signaling pathway	2	0.0367	RCHY1, TSC2
Ribosome	2	0.039	UBA52, RPL13A
mRNA surveillance pathway	2	0.039	SYMPK, PABPC1

**Down-regulated pathway names**			

Spliceosome	7	9.18E-06	RBM17, PRPF3, SNRNP40, RBNP40, RBM25, PLRG1, SRSF1, PRPF40A,
Adipocytokine signaling pathway	3	0.0173	PRKAG2, CAMKK2, PRKAB2
RNA degradation	3	0.0173	EXOSC2, CNOT10, EXOSC10
Hypertrophic cardiomyopathy (HCM)	3	0.0202	PRKAG2, PRKAB2, TPM3,
RNA polymerase	2	0.0223	POLR3GL, POLR1B
Pathways in cancer	5	0.0223	LAMA4, RUNX1, RARA, TPM3, MITF
Base excision repair	2	0.0223	HMGB1, MBD4
Lysosome	3	0.0293	LGMN, CD63, MANBA
Osteoclast differentiation	3	0.0300	CYLD, MAP3K7, MITF
RNA transport	3	0.0404	TGS1, NMD3, EEF1A1
Acute myeloid leukemia	2	0.0404	RUNX1, RARA
Purine metabolism	3	0.0423	POLR3GL, POLR1B, PDE4B

Cellular components associated with the genes down-regulated in the lambs in the CORT group compared to control lambs were macromolecular processes, and intracellular parts. These included AMP-activated protein kinase complex (2 genes; adj. *P* = 8.30E-03), Ada2/Gcn5/Ada3 transcription activator complex (2 genes; adj. *P* = 3.09E-02), sarcoplasmic reticulum (3 genes, *p* = 4.17E-02), exosome (2 genes, *P* = 4.72E-02), autophagic vacuole membrane (2 genes, adj. *P* = 4.10E-02) spliceosome complex (6 genes; adj. *P* = 8.00E-03), and contractile fibers (5 genes, *p* = 4.72E-02). Also associated with downregulated genes in CORT lambs compared to Control lambs were the nucleus and chromosome, including the nucleolus (32 genes; adj. *P* = 9.39E-06), nuclear speck (7 genes; adj. *P* = 1.40E-03), and chromatin (7 genes, *p* = 4.72E-2). The major molecular functions associated with downregulated genes in the CORT lambs compared to Control lambs were protein kinase binding (11 genes; adj. *P* = 1.64E-02) and AMP-activated protein kinase activity (2 genes; adj. *P* = 4.59E-02). Cellular metabolic processes (95 genes) and macromolecular catabolic processes (84 genes) were the major biological processes associated with downregulated genes. More specifically, macromolecule catabolic process (19 genes; adj. *P* = 4.47E-02), mRNA splicing via spliceosome (8 genes; adj. *P* = 4.47E-02) and dosage compensation by inactivation of X chromosome (2 genes; adj. *P* = 4.47E-02) were identified as overrepresented biological processes that were downregulated by the maternal cortisol treatment. KEGG pathways implicated by the downregulated genes (Table 2) were the spliceosome, adipocytokine signaling, RNA degradation, hypertrophic cardiomyopathy, RNA polymerase, pathways in cancer, base excision repair, lysosome, osteoclast differentiation, RNA transport, acute myeloid leukemia, and purine metabolism.

**FIGURE 2:**
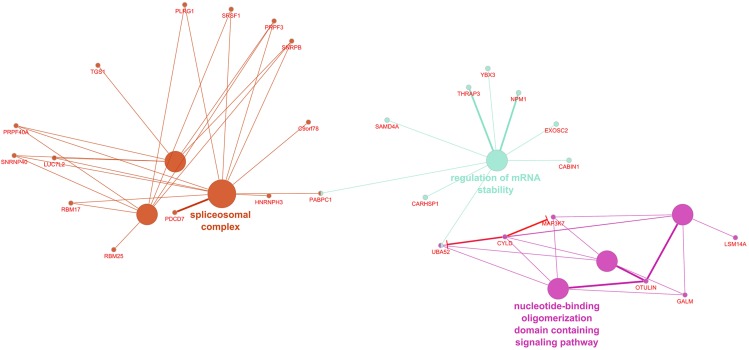
Enriched ClueGO gene network from the interventricular septum. Similar gene ontology or KEGG pathway terms are clustered by color and the representative term for those clusters are presented. Gene names shown in red; Red lines with bars represent interactions involved in gene repression.

ClueGO recognized 255 differentially expressed genes in the IVS, of which 252 had functional annotations. The 252 genes were significantly associated with 10 gene ontology terms or KEGG pathways and further organized into six groups, which were used to create an enriched network based on gene-gene interactions (Figure 2). The group with the most terms associated to it was the spliceosomal complex.

### Genes Altered by Maternal Cortisol Infusion in the Epicardial Fat of the Postnatal Lamb

Modeling of the 93 upregulated genes by prior maternal cortisol in the epicardial adipose of the CORT lambs compared to Control lambs (Supplementary Table 3) did not identify any significant biological processes or molecular function. There were also no KEGG pathways identified as significantly represented by upregulated genes. This is likely because of the relatively small number of upregulated genes. Analysis using the Bingo plugin of Cytoscape, resulted in identification of blood vessel development, angiogenesis and negative regulation of endothelial cell differentiation as the upregulated processes in epicardial fat of CORT lambs compared to Control lambs. These pathways included increased expression of NOTCH3, NOTCH4, KDR, FLT1, MEOX1, and MMP14. However, each of these genes was only modestly upregulated, approximately 20–30%; only GBP2 (guanylate binding protein 2) was upregulated more than 2-fold. Together these indicate possible upregulation of blood vessel formation in the EAT of lambs of cortisol-treated ewes compared to the controls.

There were 176 genes with decreased expression in the EAT of the 2-week-old CORT lambs after prior maternal cortisol infusion (Supplementary Table 3). The mitochondrion was the cellular compartment most significantly associated with the downregulated genes in the CORT lambs compared to Control lambs (41 genes, 1.9E-05). Catalytic activity and oxido-reductase activity were identified as the molecular functions overrepresented in the downregulated genes in the CORT lambs compared to Control lambs: these pathways included cytochrome c oxidase activity (3 genes encoding subunits of this protein, *p* = 4.66E-02), superoxide dismutase activity (2 genes encoding SOD, *p* = 3.26E-02), oxidoreductase activity, acting on the CH-CH group of donors, NAD or NADP as acceptor (3 genes, *p* = 4.66E-02), enoyl-CoA hydratase activity (2 genes, *p* = 4.66E-02), and hydroxymethylglutaryl-CoA synthase (2 genes, *p* = 2.37E-02). The biological processes identified for the downregulated genes in CORT lambs were associated with small molecule metabolic processes (47 genes), including lipid metabolic processes (27 genes) and with oxido-reduction (18 genes); more specifically the processes were regulation of fatty acid metabolic processes (6 genes, *p* = 3.68E-02), cholesterol metabolic processes (5 genes, *p* = 4.83E-02), respiratory electron transport chain (7 genes, *p* = 1.62E-02) and coenzyme A metabolic processes (4 genes, *p* = 1.62E-2).

KEGG pathways associated with the differentially down-regulated genes in the CORT lambs compared to the Control lambs are pathways related to metabolism e.g., butanoate, propanoate, fructose and mannose, fatty acid elongation, peroxisome, TCA cycle, ketone bodies, and mitochondria (e.g., Parkinson’s disease, Huntington’s disease), with some overlap between these pathways (Table 3). Bingo analysis suggested that the downregulated genes also contained an overrepresentation of genes involved in iron ion homeostasis.

**TABLE 3:** KEGG pathways significantly overrepresented in downregulated genes in EAT of 2-week-old lambs from cortisol-treated (0.5 mg/kg/day) (*n* = 9 lambs, 4 male and 5 female) compared to lambs of control ewes (*n* = 7, 3 male and 4 female).

**Down-regulated pathway names**	**#Genes**	***P*-value**	**Genes**
Metabolic pathways	32	5.6E-05	HSD17B1, COX5B, COX6C, COX6B1, GK, HK1, ACACB, UCK1, CMBL, NDUFAB1, PIGP, EPHX2, ADA, HMGCS1, HMGCS2, TMZSF2, DLD, HADHB, POLR1B, GMDS, DTYMK, FBP2, PANK1, ECHS1, DLST, CRLS1, ALDH5A1, ALDH1A1, PON3, ACLY, MLYCD, PAFAH1B2
Peroxisome	6	0.004	MLYCD, PECR, SOD1, SOD2 PECR, PEX12, EPHX2
Butanoate metabolism	4	0.004	HMGCS1, HMGCS2, ECHS1, ALDH5A1
Huntington’s disease	8	0.004	TFAM, SOD1, SOD2, VDAC1, COX5B, COX6C, COX6B1, NDUFAB1
Parkinson’s disease	6	0.006	COX5B, COX6C, COX6B1, VDAC1, NDUFAB1, UBE2G2
Valine, leucine and isoleucine degradation	5	0.006	HMGCS1, HMHCS2, HADHB, DLD, ECHS1
Citrate cycle (TCA cycle)	3	0.030	DLST, DLD, ACLY
Propanoate metabolism	3	0.036	MLYCD, ACACB, ECHS1
Synthesis and degradation of ketone bodies	2	0.036	HMGCS1, HMHCS2
Fatty acid elongation in mitochondria	2	0.036	ECHS1, HADHB
Terpenoid backbone biosynthesis	2	0.046	HMGCS1, HMHCS2
Fructose and mannose metabolism	3	0.046	HK1, GMDS, FBP2

**FIGURE 3:**
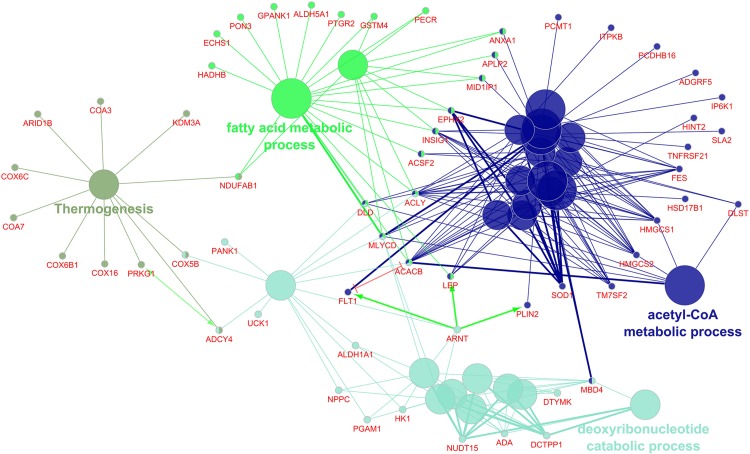
Enriched ClueGO gene network from the epicardial adipose tissue. Similar gene ontology or KEGG pathway terms are clustered by color and the representative term for those clusters are presented. Gene names shown in red; Green lines with arrows represent gene activation interactions; Red lines with bars represent interactions involved in gene repression.

The 269 differentially regulated EAT genes were entered into ClueGO; 259 had functional annotations. The 259 genes were associated with 27 gene ontology terms or KEGG pathways and further organized into 4 groups that were used to create an enriched network based on gene-gene interactions. The most significant term from each group was used as the representative term (Figure 3). The group with the most terms associated to it was the acetyl-CoA metabolic process (7 genes, *p* = 6.38E-04).

**FIGURE 4:**
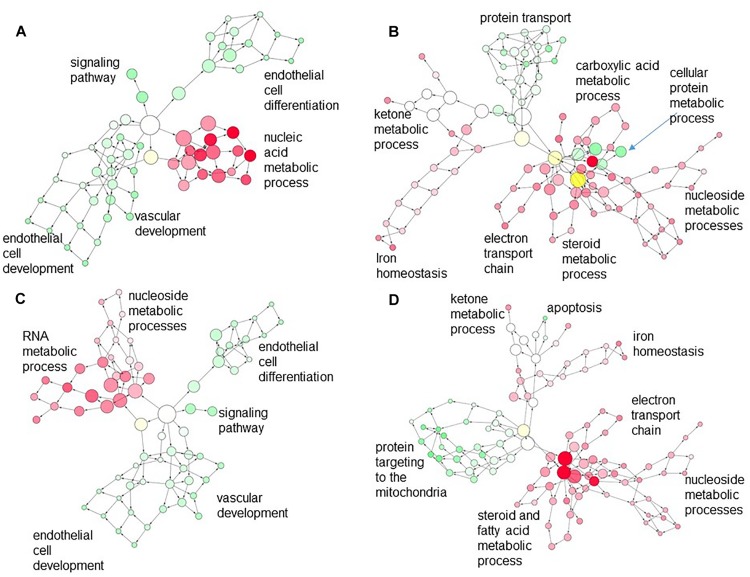
Merged network created in Cytoscape depicting interaction of the gene ontology (GO) terms associated with **(A)** down-regulated genes in the LV (red) and upregulated genes in the EAT (green); **(B)** upregulated genes in the LV (green) with downregulated genes in the EAT (red); **(C)** down-regulated genes in the IVS (red) with upregulated genes in the EAT (green); **(D)** upregulated genes in the IVS (green) with downregulated genes in the EAT (red). Each circle is a node that corresponds to a GO term. Nodes size is proportional to the number of genes associated with that GO term. Nodes are connected by edges; edge length is inversely proportional to the degree of association of the connected nodes. Density of the color in the nodes is proportional to the level of statistical significance. Higher order (most specific) GO terms appear at the periphery of the network.

Analysis of overlap of networks represented by differentially expressed genes in CORT lambs across the tissues showed relatively few genes were commonly regulated between EAT compared to either LV (14 genes) or IVS (17 genes), with only very general GO terms such as biological process or cell process associated with these commonly regulated genes. However, when we looked at the networks represented by genes differentially regulated in CORT lambs compared to Control lambs but regulated in the opposite direction in the different tissues, we found an association of some nodes between downregulated genes related to metabolic processes such as carboxylic acid, steroid or fatty acid metabolism and electron transport chain pathways in the EAT and upregulated genes related to cellular protein metabolic processes or protein transport pathways in the LV or related to protein targeting to mitochondria in IVS in CORT lambs (Figures 4B,D). In contrast the few upregulated networks in EAT did not associate with differentially downregulated genes in the LV or IVS in CORT lambs (Figures 4A,C).

## Discussion

As noted in our previous publication regarding these lambs, the majority of lambs of ewes treated with cortisol only at night survived the birth process, but at 2 weeks of life we noted a relative thickening of the left ventricular free wall. An increase in cardiac mass or wall thickness has also been previously observed in our studies with continuous maternal infusion of cortisol, which produces double the daily dose of that used in this study (Antolic et al., 2017), as well as in other models with dexamethasone treatment in sheep (Dodic et al., 2001). In the sheep heart, the cardiomyocytes are terminally differentiated by the time of birth (Jonker et al., 2007b). We do not know if the LV wall thickening occurred *in utero* during the cortisol exposure or only after birth; we also do not have data to determine if this occurs in response to higher arterial pressures postnatally in these lambs. With the higher dose of cortisol to the ewe, there was an increase in collagen deposition in the heart, and also an upregulation of the SMAD and TGFβ pathway (Antolic et al., 2019); the administration of 1 mg/kg/d to the ewe did not increase mean arterial pressure in the fetuses. However, in this current study in lambs, our transcriptomics do not model proliferation, nor do they indicate an increase in SMAD/TGFβ. We predict that the growth in these hearts is hypertrophic growth, and that any proliferation is that of fibroblasts or of endothelial cells.

Reports in human infants about the effects of *in utero* cortisol exposure are more conflicting; although postnatal steroid treatment in preterm infants appears to cause left ventricular hypertrophy during the period of therapy, other reports suggest that prenatal glucocorticoids might have either transitory or no effect (Mildenhall et al., 2006, 2009). It has been suggested that the observed hypertrophy may occur in a subset in which mothers also had gestational diabetes; which independently increases the incidence of ventricular enlargement in the newborn (Demiroren et al., 2005; Aman et al., 2011; Garcia-Flores et al., 2011). In our studies, we found that the lambs of the cortisol- treated ewes had increased glucose to insulin ratio compared to lambs of control ewes (Antolic et al., 2015), which may contribute to the mild hypertrophy we observed in these lambs.

Interestingly, in prior cohorts in which ewes were infused continuously with cortisol, producing high levels throughout the 24 h cycle, we have seen high rates of stillbirth (Keller-Wood et al., 2014), and transcriptomic modeling has suggested that the normal progression in patterns of gene transcription are altered by the maternal cortisol exposure (Richards et al., 2014; Antolic et al., 2019). In the model used here, we were able to study newborn lambs to 2 weeks of age, and use transcriptomic modeling to infer pathways altered by the effects of elevated maternal cortisol that should be further investigated in the neonate or as the offspring ages. The present study includes tissue samples that are non-homogeneous; in the left ventricular free wall and septum, this includes cardiomyocytes, Purkinje fibers, fibroblasts, endothelial cells and other components of the vascular walls. In the epicardial fat, in addition to adipose cells, there are also vascular cells and some fibroblasts. Interestingly, the transcriptomic modeling did not indicate disruption in gene expression in pathways critical for oxidative metabolism within IVS or LV as the major effect of the exposure. Although both metabolic pathways and insulin signaling were identified as KEGG pathways overrepresented by genes with increased expression in the LV in the CORT lambs as compared to the Control lambs, other metabolic pathways were associated with downregulated genes in CORT lambs, and there were few genes and no pathways that specifically relate to TCA cycle function, mitochondrial energetics, or fatty acid or glucose oxidation in the LV, nor in the IVS. This is in contrast to our transcriptomic model in the LV and IVS of the fetus at term after the higher dose, continuous maternal infusion of cortisol during the same period of gestation. In the present cohort, in both LV and IVS, pathways related to RNA transport, and RNA degradation were associated with downregulated genes in CORT lambs, and ubiquitin-mediated proteolysis and protein targeting to the mitochondria were identified as upregulated pathways in CORT lambs as compared to Control lambs. This suggests that protein synthesis and degradation may both be impacted in the lambs. In the IVS G1 to S phase transition was identified as a downregulated KEGG pathway in CORT lambs as compared to Control lambs, consistent with upregulation of genes associated with the ribosome and with endoplasmic reticulum protein processing, and suggesting cells remain in the G1 phase longer, which would be consistent with non-proliferative growth or with DNA repair, The AMPK pathway and adipocytokine signaling pathways were modeled as downregulated in IVS of CORT lambs compared to Control lambs (AMPK subunits: PRKAG2, PRKAB2, and CAMKK were downregulated). AMPK suppresses proliferative growth, and increases fatty acid oxidation, but also stimulates autophagy (Mihaylova and Shaw, 2011). Because our samples include cardiomyocytes, fibroblasts, endothelial cells and other cell types, it is not possible to determine in which cell type growth or autophagy might be altered, however, the angiogenic pathway was not identified as a pathway overrepresented by the differentially expressed genes, so that the model does not suggest any alterations in angiogenesis in the LV or IVS. Overall, the transcriptomic model is more consistent with structural remodeling in the offspring heart rather than disruption of energetics and oxidation of fatty acids, and suggests that there may be proliferation of non-myocyte cell types such as fibroblasts. Thus the study indicates that longer term studies of the offspring as they age should focus on cardiac remodeling and hypertrophy.

In EAT and LV there are indications of a functional intersection in differentially regulated pathways. Using Cytoscape, we can visualize the intersection of the networks modeled by the differentially regulated genes. In the case of LV and EAT, there appears to be a clustering of nodes (representing genes) in LV involved in cellular macromolecular and protein metabolic processes, which are upregulated in LV, with nodes in EAT for ketoacids and fatty acid metabolism, which are downregulated (Figure 4B). In our Webgestalt analysis, the models found that in both LV and EAT pathways associated with fatty acids were identified as overrepresented by the differentially regulated genes. In EAT, the pathways for fatty acid elongation (PECR, ECHS1, HADHB, and MLYCD), and ketone production (HMGCS1 and HMGCS2, encoding HMG-CoA synthase) were identified from downregulated genes. Interestingly, peroxisome was identified as a pathway with increased gene expression in LV and decreased in EAT. Peroxisomes are important for catabolism of long chain fatty acids and for production of the plasmalogens which are used in myelination, as structural phospholipids and to protect against oxidative stress, but not as substrates for mitochondrial oxidative phosphorylation (Dudda et al., 1996; Brosche and Platt, 1998; Sindelar et al., 1999). In EAT the peroxisomal genes downregulated were associated with responses to oxidative stress (SOD1, SOD2, encoding superoxide dismutases, and EPHX2, encoding epoxide hydrolase). The peroxisomal pathway genes upregulated in the LV were associated with fatty acid production [PEX13, important in peroxisomal biogenesis (Maxwell et al., 2003); SLC27A, acyl CoA synthetase [Reviewed in Soupene and Kuypers (2008)]; and ACACB, catalyzing production of malonyl CoA, a negative regulator of acyl CoA uptake (Brownsey et al., 2006)]. In a study in our laboratory in newborn lambs born to ewes treated continuously with 1 mg/kg/d cortisol, cardiac levels of plasmenyl-phosphatidylcholines (plasmenyl-PC), plasmanyl-phosphatidylethanolamines (plasmanyl-PE), and cardiolipins (CL) were reduced whereas triglycerides and diglycerides were elevated (Walejko et al., 2019). Oxidized plasmalogen products are elevated in a porcine model of myocardial infarction, and are thought to play a role in protection against oxidative stress in the heart and in the brain (Schmid and Takahashi, 1968; Dudda et al., 1996; Brosche and Platt, 1998). Thus, these results suggest that the EAT in offspring hearts may be more susceptible to oxidative stress, and that the LV is perhaps upregulating adaptive responses to oxidative stress; thus the capacity of the offspring heart to respond to oxidative challenge would be important to test in further studies in more mature offspring.

In IVS, there was also some suggestions of overlap with EAT in the functional pathways that were differentially affected by the *in utero* cortisol exposure. The intersection of GO networks was less impressive, with only negative regulation of anti-apoptosis in the IVS being closely clustered to downregulation of genes associated with metabolic changes in the EAT. There is some suggestion that triglyceride uptake and production is altered; increased expression of DGAT2, encoding an enzyme responsible for *de novo* synthesis of triglycerides, as well as LSR, encoding the protein responsible for uptake of triglycerides, was identified in the IVS. In our previous study of the newborn cardiac metabolome after maternal cortisol exposure, we found higher levels of di and triglycerides (Walejko et al., 2019). DGAT2 expression is linked to insulin resistance in muscle fibers (Levin et al., 2007). Potentially the IVS may ectopically deposit triglycerides made from the precursor metabolites that should have been stored in the EAT but were not, due to the relative immaturity of the EAT adipocytes. Ectopic deposition of “fat” in the ventricles is linked to a variety of defects of heart function [for review see (Borén et al., 2013; Mazzali et al., 2015)], so this may have important health implications. However, further studies are needed to explore a possible link between EAT dysfunction and triglyceride deposition as the animals mature.

Iron ion homeostasis was an overrepresented gene ontology in the genes downregulated in the cortisol treatment group, and this is consistent with the role of iron in controlling levels of reactive oxygen species in mitochondria during respiration. The gene most significantly reduced by maternal cortisol treatment in the microarray analyses of EAT was lactotransferrin (LTF), or lactoferrin, one of the genes included in the iron ion homeostasis category modeled. However, iron also plays a role in adipogenesis, lipolysis, lipid peroxidation, insulin-mediated glucose uptake, and mitochondrial biogenesis (Rumberger et al., 2004; Green et al., 2006; Moreno-Navarrete et al., 2014a). The macrophages of adipose depots recycle iron for proper adipocyte function, but also manipulate storage during inflammation and resolution of inflammation (Hubler et al., 2015). Thus, although maternal cortisol treatment appears to affect iron homeostasis in EAT, the causes and consequences of this are unclear from our data. The expression of genes encoding ATP citrate lyase (ACLY) and adipose differentiation-related protein (PLIN2) were reduced in expression in the EAT of the cortisol treatment group. ACLY produces acetyl CoA for lipogenesis and cholesterol genesis, and PLIN2 is a marker of lipid vacuoles of mature adipocytes. Knockdown of LTF decrease adipogenic, lipogenic and insulin signaling-related gene expression in pre-adipocytes (Moreno-Navarrete et al., 2014b) and supplementation promotes lipolysis in cultured adipocytes (Ikoma-Seki et al., 2015), and endogenous lactoferrin, produced by adipocytes, is necessary for the differentiation of preadipocytes to adipocytes (Moreno-Navarrete et al., 2013). These gene expression changes suggest that the EAT of the treated lambs is still proliferating and or relatively undifferentiated compared to the EAT of lambs of untreated ewes. This is complemented by the continued angiogenesis modeled in the cortisol treatment group, together these imply continued growth of the EAT depot. We were technically unable to measure whether there was an increase in the size of this adipose tissue depot due to hyperplasia following maternal cortisol treatment. These data suggest a larger depot might be more evident in older animals than those used in this study when the depot has completely formed. A larger EAT depot could have important health consequences. For example, the thickness of human EAT is correlated at only 8 years of age with increased risk of higher corrected-BMI and metabolic syndrome (Barbaro et al., 2016), and the volume and density of EAT is associated with cardiovascular disease including hypertension (Börekçi et al., 2015), resistant hypertension (Erdogan et al., 2016), myocardial ischemia (Hell et al., 2016) and coronary artery calcification (Gauss et al., 2015).

The effects of maternal cortisol are substantially different than those of maternal nutrition (either over or under) or placental restriction, which effect changes in leptin, adiponectin, PPARγ signaling, and IGFs that parallel the plane of nutrient supply to the fetus (Muhlhausler et al., 2007; Duffield et al., 2008, 2009), suggesting that this effect is not secondary to a limitation in substrate ability to the heart *in utero*. Our results do suggest long-lasting effects of the maternal hypercortisolemia on the postnatal heart, and suggest that alterations in epicardial adipose maturation and function may contribute to changes in cardiac structure and function. The transcriptomic model is consistent with the phenotype of the lambs; they were healthy and grew normally, and had minor cardiac wall thickening (Antolic et al., 2015).

The importance of glucocorticoids for organ maturation in late gestation (Silver, 1990; Liggins, 1994; Fowden and Silver, 1995; Phillips et al., 1997; Rog-Zielinska et al., 2013; Agnew et al., 2018) is most evident in preterm babies, in whom antenatal glucocorticoid therapy is essential for improving lung maturation; however, this life-saving intervention may also increase the risk for developing cardiovascular disease later in life (Benediktsson et al., 1993; Seckl, 2004; Dalziel et al., 2005; McKinlay et al., 2015). Accumulating evidence now shows that glucocorticoids are also necessary for cardiac maturation, and that excessive *in utero* exposure to glucocorticoids can negatively impact the maturational process. Studies from our lab indicate that elevations in maternal cortisol concentration throughout late gestation alters the normal trajectory of cardiac gene expression in the ovine fetus (Richards et al., 2014; Antolic et al., 2019), which persists into postnatal development. Although the underlying mechanism(s) is still under investigation, it appears to involve multiple signaling pathways (particularly those involved in cardiac metabolism), transcription factors, and protein abundance and activity. As a consequence, perinatal exposure to glucocorticoids may program the cardiac transcriptome, leaving individuals exposed during that developmental window susceptible to cardiovascular disease.

## Data Availability

The datasets generated for this study can be found in the National Center for Biotechnology Information’s and the Gene Expression Omnibus, GSE131537 and GSE119254.

## Ethics Statement

This study was carried out in accordance with the recommendations of the NIH Guide for the Care and Use of Laboratory Animals. The protocol was approved by the University of Florida Institutional Care and Use Committee.

## Author Contributions

MK-W and ER designed the study. AA and ER carried out the array analysis. All authors analyzed and interpreted the data, and drafted and approved the final version of the manuscript. AA and MK-W prepared the figures.

## Conflict of Interest Statement

The authors declare that the research was conducted in the absence of any commercial or financial relationships that could be construed as a potential conflict of interest.
