# Supplementary material for: A Transcriptomic Model of Postnatal Cardiac Effects of Prenatal Maternal Cortisol Excess in Sheep
Source: Front Physiol. 2019 Jul 3;10:816. doi: 10.3389/fphys.2019.00816 (PMC6616147; doi:10.3389/fphys.2019.00816)
Supplement: Supplementary file 1 [file Table_1.pdf]

Supplemental Table 1: Differentially expressed genes ($p<0.05$) in Left Ventricle of 2week old lambs after maternal cortisol treatment

Fold changes are expressed as values in lambs of cortisol-treated ewes compared to control lambs

Official Symbol	Gene Name	Fold Change	P.Value
GBP2	guanylate binding protein 2, interferon-inducible	2.41	0.0510
RGS2	regulator of G-protein signaling 2	1.55	0.0051
INHA	inhibin, alpha	1.51	0.0306
MYL6B	myosin, light chain 6B	1.43	0.0115
CINP	cyclin-dependent kinase 2-interacting protein	1.37	0.0138
C7orf23	chromosome 7 open reading frame 23	1.32	0.0414
TOR3A	torsin family 3, member A	1.31	0.0477
MOBKL1A	MOB1, Mps One Binder kinase activator-like 1A (yeast)	1.31	0.0219
FAM108C1	family with sequence similarity 108, member C1	1.29	0.0058
TAF2	TATA box binding protein (TBP)-associated factor 2	1.28	0.0023
PEG10	paternally expressed 10	1.27	0.0121
GZMB	granzyme B	1.27	0.0115
ING3	inhibitor of growth family, member 3	1.26	0.0158
TMCO6	transmembrane and coiled-coil domains 6	1.26	0.0008
FBXO31	F-box protein 31	1.25	0.0126
ALG1	asparagine-linked glycosylation 1	1.25	0.0128
DDX3X	DEAD (Asp-Glu-Ala-Asp) box polypeptide 3, X-linked	1.24	0.0528
UBR1	ubiquitin protein ligase E3 component n-recognin 1	1.24	0.0439
FKBP4	FK506 binding protein 4, 59kDa	1.23	0.0107
MFN2	mitofusin 2	1.23	0.0485
FZD6	frizzled homolog 6 (Drosophila)	1.23	0.0048
CFL1	cofilin 1 (non-muscle)	1.23	0.0314
REXO4	REX4, RNA exonuclease 4 homolog (S. cerevisiae)	1.22	0.0042
SLC27A2	solute carrier family 27 (fatty acid transporter), member 2	1.21	0.0238
TIMM8A	translocase of inner mitochondrial membrane 8 homolog A	1.21	0.0156
NME2	NME/NM23 Nucleoside Diphosphate Kinase 2	1.21	0.0175
DHRS11	dehydrogenase/reductase (SDR family) member 11	1.21	0.0085
TRAPPC6B	trafficking protein particle complex 6B	1.20	0.0276
ABP1	amiloride binding protein 1 (amine oxidase (copper-containing))	1.20	0.0440
ACACB	acetyl-Coenzyme A carboxylase beta	1.20	0.0231
HERPUD1	homocysteine-inducible, endoplasmic reticulum stress-inducible, ubiquitin-like domain member 1	1.20	0.0267
ANAPC13	anaphase promoting complex subunit 13	1.19	0.0105
BMI1	BMI1 polycomb ring finger oncogene	1.19	0.0534
ASNA1	arsA arsenite transporter, ATP-binding, homolog 1	1.19	0.0373

EGR3	early growth response 3	1.18	0.0245
MRPL21	mitochondrial ribosomal protein L21	1.18	0.0102
ACAP1	ArfGAP with coiled-coil, ankyrin repeat and PH domains 1	1.18	0.0286
SULF2	sulfatase 2	1.18	0.0493
PPID	peptidylprolyl isomerase D	1.18	0.0524
TCEB2	transcription elongation factor B (SIII), polypeptide 2 (18kDa, elongin B)	1.18	0.0022
WSB2	WD repeat and SOCS box-containing 2	1.17	0.0129
DSCC1	defective in sister chromatid cohesion 1 homolog (S. cerevisiae)	1.17	0.0190
RCHY1	ring finger and CHY zinc finger domain containing 1	1.17	0.0030
SLC38A5	solute carrier family 38, member 5	1.17	0.0510
CDK18	PCTAIRE protein kinase 3	1.17	0.0329
SNTB2	syntrophin, beta 2 (dystrophin-associated protein A1, 59kDa, basic component 2)	1.17	0.0371
BRWD1	bromodomain and WD repeat domain containing 1	1.17	0.0538
SF3B3	splicing factor 3b, subunit 3, 130kDa	1.17	0.0144
NR3C1	nuclear receptor subfamily 3, group C, member 1	1.16	0.0363
KIDINS220	kinase D-interacting substrate, 220kDa	1.16	0.0158
NRP1	neuropilin 1	1.16	0.0181
DUSP6	dual specificity phosphatase 6	1.16	0.0498
SREBF1	sterol regulatory element binding transcription factor 1	1.16	0.0244
LNX2	ligand of numb-protein X 2	1.16	0.0369
ANXA6	annexin A6	1.16	0.0523
CBR4	carbonyl reductase 4	1.16	0.0312
SDCCAG8	serologically defined colon cancer antigen 8	1.15	0.0102
GABPA	GA binding protein transcription factor, alpha subunit 60kDa	1.15	0.0198
GTF2H5	general transcription factor IIH, polypeptide 5	1.15	0.0416
MRPL19	mitochondrial ribosomal protein L19	1.15	0.0359
ATP5S	ATP synthase, H+ transporting, mitochondrial F0 complex, subunit s	1.15	0.0314
MRPL48	mitochondrial ribosomal protein L48	1.15	0.0112
PIN1	peptidylprolyl cis/trans isomerase, NIMA-interacting 1	1.15	0.0335
HDGF	hepatoma-derived growth factor (high-mobility group protein 1-like)	1.15	0.0207
C19orf2	chromosome 19 open reading frame 2	1.15	0.0432
UCKL1	uridine-cytidine kinase 1-like 1	1.15	0.0237
TOMM40	translocase of outer mitochondrial membrane 40 homolog	1.14	0.0308
TOMM5	translocase of outer mitochondrial membrane 5 homolog	1.14	0.0246
CISD1	CDGSH iron sulfur domain 1	1.14	0.0321
LRRC47	leucine rich repeat containing 47	1.14	0.0180

ELAVL1	ELAV (embryonic lethal, abnormal vision, Drosophila)-like 1 (Hu antigen R)	1.14	0.0088
DNAJC11	DnaJ (Hsp40) homolog, subfamily C, member 11	1.14	0.0411
CNOT7	CCR4-NOT transcription complex, subunit 7	1.13	0.0468
ZFAND5	similar to zinc finger, AN1-type domain 5; zinc finger, AN1-type domain 5	1.13	0.0186
IPO5	importin 5	1.13	0.0442
KLHL9	kelch-like 9 (Drosophila)	1.13	0.0508
FNIP1	folliculin interacting protein 1	1.13	0.0502
KCTD20	potassium channel tetramerisation domain containing 20	1.13	0.0261
SLC7A1	solute carrier family 7 (cationic amino acid transporter, y+ system), member 1	1.13	0.0418
PEX13	peroxisomal biogenesis factor 13	1.12	0.0531
MMAB	methylmalonic aciduria (cobalamin deficiency) cblB type	1.12	0.0403
NUMB	numb homolog (Drosophila)	1.12	0.0521
C19orf43	chromosome 19 open reading frame 43	1.12	0.0272
ARHGAP29	Rho GTPase activating protein 29	1.12	0.0468
ZNF292	zinc finger protein 292	1.12	0.0462
HPRT1	hypoxanthine phosphoribosyltransferase 1	1.11	0.0231
MRPS10	mitochondrial ribosomal protein S10	1.11	0.0296
ZNF414	zinc finger protein 414	1.11	0.0108
PHOSPHO2	phosphatase, orphan 2	1.11	0.0507
BIRC6	baculoviral IAP repeat-containing 6	1.11	0.0216
CALM2	calmodulin 2 (phosphorylase kinase, delta)	1.11	0.0542
FOXS1	forkhead box S1	1.11	0.0108
PTOV1	prostate tumor overexpressed 1	1.11	0.0159
CTDSPL2	CTD (carboxy-terminal domain, RNA polymerase II, polypeptide A) small phosphatase like 2	1.10	0.0387
CTDSP1	CTD (carboxy-terminal domain, RNA polymerase II, polypeptide A) small phosphatase 1	1.10	0.0385
PPP2R4	protein phosphatase 2A activator, regulatory subunit 4	1.10	0.0300
RB1CC1	RB1-inducible coiled-coil 1	1.10	0.0496
C9orf80	chromosome 9 open reading frame 80	1.10	0.0321
ZBTB11	zinc finger and BTB domain containing 11	1.10	0.0526
UBA2	ubiquitin-like modifier activating enzyme 2	1.10	0.0324
PSMG2	proteasome (prosome, macropain) assembly chaperone 2	1.10	0.0437
CHMP2B	chromatin modifying protein 2B	1.10	0.0360
ZFAND6	zinc finger, AN1-type domain 6	1.10	0.0426
MAP2	microtubule-associated protein 2	1.10	0.0540
SRPK2	SFRS protein kinase 2	1.10	0.0375
PPM1A	protein phosphatase 1A	1.09	0.0407

USP39	ubiquitin specific peptidase 39	1.09	0.0464
AGPS	alkylglycerone phosphate synthase	1.09	0.0243
TICAM2	toll-like receptor adaptor molecule 2	1.09	0.0539
ETF1	eukaryotic translation termination factor 1	1.09	0.0482
PIP4K2A	phosphatidylinositol-5-phosphate 4-kinase, type II, alpha	1.09	0.0354
SNX3	sorting nexin 3	1.09	0.0433
LUZP6	myotrophin; leucine zipper protein 6	1.08	0.0484
STARD13	StAR-related lipid transfer (START) domain containing 13	1.07	0.0496
C1orf212	chromosome 1 open reading frame 212	0.93	0.0538
FAM175B	family with sequence similarity 175, member B	0.92	0.0438
HNRNPUL1	heterogeneous nuclear ribonucleoprotein U-like 1	0.92	0.0502
RPS2	ribosomal protein S2	0.92	0.0313
SRSF4	serine and arginine rich splicing factor 4	0.92	0.0386
GART	phosphoribosylglycinamide formyltransferase, phosphoribosylglycinamide synthetase, phosphoribosylaminoimidazole synthetase	0.91	0.0451
SNAPIN	SNAP-associated protein	0.91	0.0543
CLOCK	clock homolog (mouse)	0.91	0.0130
UBTD1	ubiquitin domain containing 1	0.91	0.0278
THYN1	thymocyte nuclear protein 1	0.91	0.0381
MESDC2	mesoderm development candidate 2	0.91	0.0514
SRL	sarcalumenin	0.91	0.0157
WAPAL	wings apart-like homolog (Drosophila)	0.91	0.0231
SAP30BP	SAP30 binding protein	0.90	0.0538
GLT8D1	glycosyltransferase 8 domain containing 1	0.90	0.0323
CYBA	cytochrome b-245, alpha polypeptide	0.90	0.0337
SDF2	stromal cell-derived factor 2	0.90	0.0209
DENND4B	DENN/MADD domain containing 4B	0.90	0.0442
NUMA1	nuclear mitotic apparatus protein 1	0.90	0.0538
SLC50A1	solute carrier family 50 member 1	0.90	0.0517
IFT46	intraflagellar transport 46	0.90	0.0306
TAF10	TAF10 RNA polymerase II, TATA box binding protein (TBP)-associated factor, 30kDa	0.90	0.0433
NTHL1	nth endonuclease III-like 1	0.90	0.0154
CC2D1B	coiled-coil and C2 domain containing 1B	0.90	0.0286
DDRGK1	DDRGK domain containing 1	0.90	0.0095
RPS8	ribosomal protein S8	0.90	0.0280
CHAF1A	chromatin assembly factor 1, subunit A (p150)	0.90	0.0327
RUFY3	RUN and FYVE domain containing 3	0.90	0.0527
C17orf59	chromosome 17 open reading frame 59	0.90	0.0312
POP7	processing of precursor 7, ribonuclease P/MRP subunit	0.89	0.0273

TUSC2	tumor suppressor candidate 2	0.89	0.0466
ELAC1	elaC homolog 1 (E. coli)	0.89	0.0110
RFFL	ring finger and FYVE-like domain containing 1	0.89	0.0347
SREK1IP1	SREK 1 interacting protein 1	0.89	0.0373
CDK7	cyclin-dependent kinase 7	0.89	0.0427
DSCR3	Down syndrome critical region gene 3	0.89	0.0441
QPCTL	glutaminyl-peptide cyclotransferase-like	0.89	0.0268
TMEM179B	transmembrane protein 179B	0.89	0.0304
TLR6	toll-like receptor 6	0.89	0.0456
C12orf11	chromosome 12 open reading frame 11	0.89	0.0216
IL17D	interleukin 17D	0.88	0.0460
ZBTB6	zinc finger and BTB domain containing 6	0.88	0.0402
MICALL1	MICAL-like 1	0.88	0.0486
C9orf16	chromosome 9 open reading frame 16	0.88	0.0385
PPAP2C	phosphatidic acid phosphatase type 2C	0.88	0.0342
RAB11FIP5	RAB11 family interacting protein 5 (class I)	0.88	0.0508
SLC12A7	solute carrier family 12 (potassium/chloride transporters), member 7	0.88	0.0486
C15orf24	chromosome 15 open reading frame 24	0.88	0.0345
FAM3C	family with sequence similarity 3, member C	0.88	0.0281
PLCD1	phospholipase C, delta 1	0.88	0.0230
DCP2	decapping MRNA2	0.87	0.0248
RBFOX2	RNA binding FOX homolog 2	0.87	0.0029
MPG	N-methylpurine-DNA glycosylase	0.87	0.0290
KEAP1	kelch-like ECH-associated protein 1	0.87	0.0342
STAP2	signal transducing adaptor family member 2	0.87	0.0234
CREB3	cAMP responsive element binding protein 3	0.87	0.0091
TRAPPC2L	trafficking protein particle complex 2-like	0.87	0.0378
CHD4	chromodomain helicase DNA binding protein 4	0.87	0.0156
SART3	squamous cell carcinoma antigen recognized by T cells 3	0.87	0.0379
FANCG	Fanconi anemia, complementation group G	0.87	0.0048
TUBB2C	tubulin, beta 2C	0.87	0.0505
ABCB10	ATP-binding cassette, sub-family B (MDR/TAP), member 10	0.87	0.0336
C1H21ORF59		0.87	0.0420
ATP1B3	ATPase, Na+/K+ transporting, beta 3 polypeptide	0.87	0.0382
ORM1	orosomucoid 1	0.87	0.0403
N6AMT2	N-6 adenine-specific DNA methyltransferase 2 (putative)	0.87	0.0389
PGRMC2	progesterone receptor membrane component 2	0.86	0.0130
EIF4G1	eukaryotic translation initiation factor 4 gamma, 1	0.86	0.0105
THRB	thyroid hormone receptor, beta	0.86	0.0249
ST6GAL1	ST6 beta-galactosamide alpha-2,6-sialyltranferase 1	0.86	0.0199

NARS	asparaginyl-tRNA synthetase	0.86	0.0382
ATG16L1	ATG16 autophagy related 16-like 1 (S. cerevisiae)	0.86	0.0542
CYP27A1	cytochrome P450, family 27, subfamily A, polypeptide 1	0.86	0.0108
PIGT	phosphatidylinositol glycan anchor biosynthesis, class T	0.86	0.0267
EML4	echinoderm microtubule associated protein like 4	0.86	0.0507
PBXIP1	pre-B-cell leukemia homeobox interacting protein 1	0.86	0.0291
HFE	hemochromatosis	0.86	0.0038
BLCAP	bladder cancer associated protein	0.86	0.0063
TUBB2B	tubulin, beta 2B	0.86	0.0282
NPEPPS	aminopeptidase puromycin sensitive	0.86	0.0344
PICK1	protein interacting with PRKCA 1	0.86	0.0239
TUBB3	tubulin, beta 3; melanocortin 1 receptor	0.86	0.0290
SURF6	surfeit 6	0.86	0.0321
DERL1	Der1-like domain family, member 1	0.86	0.0333
IMP4	U3 small nucleolar ribonucleoprotein	0.85	0.0433
FUBP1	far upstream element (FUSE) binding protein 1	0.85	0.0509
DHCR7	7-dehydrocholesterol reductase	0.85	0.0473
PSMD7	proteasome 26S subunit, non-ATPase, 7	0.85	0.0046
OSTBETA	organic solute transporter beta	0.85	0.0087
RWDD2A	RWD domain containing 2A	0.85	0.0142
C14H11orf75		0.85	0.0178
VEZF1	vascular endothelial zinc finger 1	0.85	0.0472
ZMYND11	zinc finger, MYND domain containing 11	0.85	0.0277
EXOSC2	exosome component 2	0.85	0.0230
EZH1	enhancer of zeste homolog 1	0.85	0.0517
LATS2	LATS, large tumor suppressor, homolog 2	0.84	0.0039
CSNK1E	casein kinase 1, epsilon	0.84	0.0384
BAX	BCL2-associated X protein	0.83	0.0066
CNOT10	CCR4-NOT transcription complex, subunit 10	0.83	0.0355
CRYL1	crystallin, lambda 1	0.83	0.0262
DKC1	dyskeratosis congenita 1, dyskerin	0.83	0.0530
NCOR1	nuclear receptor co-repressor 1	0.83	0.0402
LRSAM1	leucine rich repeat and sterile alpha motif containing 1	0.83	0.0216
MAF	MAF BZIP transcription factor	0.83	0.0059
QSOX2	quiescin Q6 sulfhydryl oxidase 2	0.83	0.0413
AKR1E2	aldo-keto reductase family 1, member C-like 2	0.83	0.0347
ADRB1	adrenergic, beta-1-, receptor	0.82	0.0135
TRIM45	tripartite motif-containing 45	0.82	0.0125
BTBD10	BTB (POZ) domain containing 10	0.81	0.0082
C20orf108	chromosome 20 open reading frame 108	0.81	0.0323
ABR	active BCR-related gene	0.81	0.0543

CCDC76	coiled-coil domain containing 76	0.81	0.0198
RAD50	RAD50 homolog (S. cerevisiae)	0.81	0.0441
PTGER3	prostaglandin E receptor 3 (subtype EP3)	0.81	0.0106
CSNK1G2	casein kinase 1, gamma 2	0.81	0.0028
DPY19L1	dpy-19-like 1 (C. elegans); similar to hCG1645499	0.81	0.0227
TMEM106C	transmembrane protein 106C	0.80	0.0096
EIF3A	eukaryotic translation initiation factor 3, subunit A	0.80	0.0338
TBC1D7	TBC1 domain family, member 7	0.80	0.0038
DDX19B	DEAD (Asp-Glu-Ala-As) box polypeptide 19B	0.80	0.0117
MYSM1	Myb-like, SWIRM and MPN domains 1	0.79	0.0062
TSPO	translocator protein (18kDa)	0.78	0.0424
GRAP	GRB2-related adaptor protein	0.78	0.0127
IWS1	IWS1 homolog (S. cerevisiae)	0.76	0.0235
LGMN	legumain	0.76	0.0088
PAPOLA	poly(A) polymerase alpha	0.76	0.0303
SCPEP1	serine carboxypeptidase 1	0.76	0.0112
CAST	calpastatin	0.75	0.0279
PDE4B	phosphodiesterase 4B, cAMP-specific	0.75	0.0269
COPB2	coatomer protein complex, subunit beta 2	0.75	0.0001
ZNF691	zinc finger protein 691	0.74	0.0015
C9orf78	chromosome 9 open reading frame 78	0.74	0.0049
GHR	growth hormone receptor	0.74	0.0027
ADD1	adducin 1 (alpha)	0.72	0.0148
NUDT16	nudix (nucleoside diphosphate linked moiety X)-type motif 16	0.71	0.0207
RBP7	retinol binding protein 7, cellular	0.70	0.0133
MYO18A	myosin XVIIIA	0.69	0.0071
TCEAL2	transcription elongation factor A (SII)-like 2	0.68	0.0115
ABCF1	ATP-binding cassette, sub-family F (GCN20), member 1	0.67	0.0106
DNAJC21	DnaJ (Hsp40) homolog, subfamily C, member 21	0.67	0.0005
SBSN	suprabasin	0.66	0.0033
XPO4	exportin 4	0.65	0.0172
TMOD3	tropomodulin 3 (ubiquitous)	0.65	0.0109
DHRS13	dehydrogenase/reductase (SDR family) member 13	0.63	0.0201
HBA1	hemoglobin, alpha 2; hemoglobin, alpha 1	0.46	0.0110
