# Supplementary material for: A Transcriptomic Model of Postnatal Cardiac Effects of Prenatal Maternal Cortisol Excess in Sheep
Source: Front Physiol. 2019 Jul 3;10:816. doi: 10.3389/fphys.2019.00816 (PMC6616147; doi:10.3389/fphys.2019.00816)
Supplement: Supplementary file 2 [file Table_2.pdf]

Supplemental Table 2: Differentially expressed genes ($p<0.05$) in Intraventricular Septum of 2week old lambs after maternal cortisol treatment

Fold changes are expressed as values in lambs of cortisol-treated ewes compared to control lambs

OFFICIAL SYMBOL	Gene Name	Fold Change	P.Value
CLCN6	chloride channel 6	4.43	0.0121
CDK2AP2	cyclin-dependent kinase 2 associated protein 2	3.43	0.0091
TM9SF2	transmembrane 9 superfamily member 2	3.24	0.0164
NDOR1	NADPH dependent diflavin oxidoreductase 1	3.19	0.0157
LSR	lipolysis stimulated lipoprotein receptor	2.95	0.0097
LSM14A	LSM14A, SCD6 homolog A (S. cerevisiae)	2.69	0.0273
DEFA6	defensin, alpha 6, Paneth cell-specific	2.67	0.0068
**DGAT2**	diacylglycerol O-acyltransferase homolog 2	2.62	0.0024
CLASP1	cytoplasmic linker associated protein 1	2.48	0.0192
TSC2	tuberous sclerosis 2	2.29	0.0085
KLHL26	kelch-like 26	2.26	0.0544
GPC1	glypican 1	2.25	0.0135
KIAA1826	KIAA1826	2.17	0.0532
CARHSP1	calcium regulated heat stable protein 1, 24kDa	2.05	0.0403
CXCL2	chemokine (C-X-C motif) ligand 2	2.05	0.0201
NAPSA	napsin A aspartic peptidase	1.94	0.0523
C19orf61	chromosome 19 open reading frame 61	1.87	0.0107
CDC42EP5	CDC42 effector protein (Rho GTPase binding) 5	1.86	0.0422
FBLN5	fibulin 5	1.81	0.0517
RGS2	regulator of G-protein signaling 2, 24kDa	1.69	0.0016
SEC24C	SEC24 family, member C	1.65	0.0276
FIZ1	FLT3-interacting zinc finger 1	1.65	0.0242
TAX1BP3	Tax1 (T-cell leukemia virus type I) binding protein 3	1.59	0.0375
MYL6B	myosin, light chain 6B	1.58	0.0153
FRK	fyn-related kinase	1.57	0.0478
NOP10	NOP10 ribonucleoprotein homolog	1.55	0.0295
IGL@	immunoglobulin lambda locus	1.54	0.0408
ATP2A3	ATPase, Ca++ transporting, ubiquitous	1.54	0.0359
USP5	ubiquitin specific peptidase 5	1.50	0.0319
MFAP2	microfibrillar-associated protein 2	1.46	0.0251
ING3	inhibitor of growth family, member 3	1.41	0.0133
C15orf48	chromosome 15 open reading frame 48	1.40	0.0106
TNFAIP8	tumor necrosis factor, alpha-induced protein 8	1.39	0.0404
RBM18	RNA binding motif protein 18	1.38	0.0521
KIF2C	kinesin family member 2C	1.37	0.0502
KCNJ1	potassium inwardly-rectifying channel, subfamily J, member 1	1.37	0.0179

UBR1	ubiquitin protein ligase E3 component n-recognin 1	1.35	0.0135
RPL13A	ribosomal protein L13a	1.35	0.0276
TCEB1	elongin C	1.33	0.0173
RNF126	ring finger protein 126	1.32	0.0236
ECI1	enoyl coenzyme A delta isomerase 1	1.32	0.0398
CBR1	carbonyl reductase 1	1.32	0.0416
ACYP1	acylphosphatase 1	1.31	0.0458
C21orf33	chromosome 21 open reading frame 33	1.31	0.0334
SPHK1	sphingosine kinase 1	1.30	0.0344
HERPUD1	homocysteine-inducible endoplasmic reticulu protein with ubiquitin-like domain	1.30	0.0056
MRPS11	mitochondrial ribosomal protein S11	1.29	0.0386
TMCO6	transmembrane and coiled-coil domains 6	1.29	0.0135
UBA52	ubiquitin A-52 residue ribosomal protein fusion product 1	1.28	0.0366
AIMP2	aminoacyl tRNA synthetase complex-interacting multifunctional protein 2	1.28	0.0037
SYMPK	symplekin	1.28	0.0340
SEC13	SEC13 homolog	1.28	0.0250
CCL14	chemokine (C-C motif) ligand 14	1.28	0.0359
TCEANC2	transcription elongation factor N-terminal and central domain containing 2	1.28	0.0326
DYNLRB1	dynein light chain roadblock-type 1	1.28	0.0468
SYS1	SYS1 Golgi-localized integral membrane protein homolog	1.27	0.0135
TSPAN4	tetraspanin 4	1.27	0.0168
PRADC1	protease associated domain containing 1	1.26	0.0531
ANAPC11	anaphase promoting complex subunit 11	1.26	0.0389
PABPC1	poly(A) binding protein cytoplasmic 1	1.26	0.0524
GRINA	glutamate ionotropic receptor N-methyl D-aspartate-associated protein 1	1.26	0.0518
SPSB1	splA/ryanodine receptor domain and SOCS box containing 1	1.26	0.0070
YPEL1	yippee-like 1	1.25	0.0503
TOMM5	translocase of outer mitochondrial membrane 5 homolog (yeast)	1.25	0.0495
HIPK3	homeodomain interacting protein kinase 3	1.25	0.0183
MPST	mercaptopyruvate sulfurtransferase	1.24	0.0085
TMEM80	transmembrane protein 80	1.24	0.0544
YIPF6	Yip1 domain family, member 6	1.23	0.0451
VPS28	vacuolar protein sorting 28	1.23	0.0115
LIMCH1	LIM and calponin homology domains 1	1.23	0.0171
FABP4	fatty acid binding protein 4	1.23	0.0087
CISD1	CDGSH iron sulfur domain 1	1.23	0.0282
KPNA4	karyopherin alpha 4	1.22	0.0530
KRTAP13-1	keratin associated protein 13-1	1.22	0.0215
DIDO1	death inducer-obliterator 1	1.22	0.0485

SNRPB	small nuclear ribonucleoprotein polypeptides B and B1	1.22	0.0403
MRPL33	mitochondrial ribosomal protein L33	1.21	0.0542
MTX1	metaxin 1	1.21	0.0513
TIMM8A	translocase of inner mitochondrial membrane 8 homolog A	1.21	0.0534
STOML2	stomatin (EPB72)-like 2	1.21	0.0209
MRPL2	mitochondrial ribosomal protein L2	1.21	0.0510
TIMM8B	similar to translocase of inner mitochondrial membrane 8 homolog B	1.21	0.0529
ECH1	enoyl Coenzyme A hydratase 1	1.21	0.0298
C6orf57	chromosome 6 open reading frame 57	1.20	0.0526
C1orf31	chromosome 1 open reading frame 31	1.20	0.0425
MRPS33	mitochondrial ribosomal protein S33	1.20	0.0237
CD52	CD52 molecule	1.20	0.0454
PLEC	plectin 1	1.19	0.0101
BRWD1	bromodomain and WD repeat domain containing 1	1.18	0.0213
PAG10	phosphoprotein membrane anchor with glycosphingolipid microdomains 1	1.18	0.0268
RCHY1	ring finger and CHY zinc finger domain containing 1	1.18	0.0286
ETNK1	ethanolamine kinase 1	1.17	0.0475
SIVA1	SIVA1, apoptosis-inducing factor	1.17	0.0340
GSTM4	glutathione S-transferase mu 4	1.17	0.0254
CEP350	centrosomal protein 350kDa	1.17	0.0498
MORN4	MORN repeat containing 4	1.17	0.0512
VAMP8	vesicle-associated membrane protein 8	1.17	0.0530
SDHAF1	succinate dehydrogenase complex assembly factor 1	1.17	0.0216
FRAT2	frequently rearranged in advanced T-cell lymphomas 2	1.16	0.0524
NRAP	nebulin-related anchoring protein	1.15	0.0405
ERP29	endoplasmic reticulum protein 29	1.14	0.0367
CABIN1	calcineurin binding protein 1	1.14	0.0171
TCEA2	transcription elongation factor A (SII), 2	1.11	0.0353
PAK2	p21 (RAC) activated kinase 2	0.90	0.0274
KAT2B	K(lysine) acetyltransferase 2B	0.89	0.0319
PRPF3	PRP3 pre-mRNA processing factor 3	0.89	0.0518
VPS35	VPS35 rotomer complex component	0.89	0.0320
EXOSC10	exosome component 10	0.89	0.0517
PRPF40A	PRP40 pre-mRNA processing factor 40 homolog A	0.89	0.0499
GRHL1	grainyhead-like 1	0.89	0.0210
SLC30A6	solute carrier family 30 member 6	0.88	0.0523
CYLD	cylindromatosis	0.88	0.0539
PLAA	phospholipase A2-activating protein	0.88	0.0407
STARD3NL	STARD3 N-terminal like	0.88	0.0343
GTF2E1	general transcription factor IIE subunit 1	0.88	0.0345
SMCHD1	structural maintenance of chromosomes flexible hinge domain containing 1	0.87	0.0336

N6AMT2	N-6 adenine-specific DNA methyltransferase 2 (putative)	0.87	0.0536
BUB3	budding uninhibited by benzimidazoles 3 homolog	0.87	0.0505
FAM105B	family with sequence similarity 105, member B	0.87	0.0387
TPM3	tropomyosin 3	0.86	0.0542
RAB2B	RAB2B, member RAS oncogene family	0.86	0.0521
HMGB1	high-mobility group box 1; high-mobility group box 1-like 10	0.86	0.0356
POLR3GL	RNA polymerase III subunit G like	0.86	0.0391
KCTD6	potassium channel tetramerisation domain containing 6	0.86	0.0470
FAM104A	family with sequence similarity 104 member A	0.86	0.0380
PBRM1	polybromo 1	0.86	0.0520
SRPRB	signal recognition particle receptor, B subunit	0.86	0.0273
PTPRM	protein tyrosine phosphatase, receptor type M	0.86	0.0253
OFD1	oral-facial-digital syndrome 1	0.86	0.0387
TGS1	trimethylguanosine synthase homolog	0.86	0.0445
RNF115	ring finger protein 115	0.85	0.0308
ZNF45	zinc finger protein 45	0.85	0.0272
RBM17	RNA binding motif protein 17	0.85	0.0456
HOMEZ	homeobox and leucine zipper encoding	0.85	0.0393
RPL38	ribosomal protein L38	0.85	0.0456
SENP1	SUMO1/sentrin specific peptidase 1	0.85	0.0434
C1GALT1C1	C1GALT1-specific chaperone 1	0.85	0.0375
POLR1B	RNA polymerase I polypeptide B	0.85	0.0396
RFWD2	ring finger and WD repeat domain 2	0.84	0.0321
MAP4K3	mitogen-activated protein kinase kinase kinase kinase 3	0.84	0.0182
LDOC1L	leucine zipper, down-regulated in cancer 1-like	0.84	0.0203
FAM175B	family with sequence similarity 175, member B	0.84	0.0218
SFXN4	sideroflexin 4	0.84	0.0318
ADAM9	ADAM metallopeptidase domain 9	0.84	0.0174
GSPT1	G1 to S phase transition 1	0.84	0.0177
TSEN15	tRNA splicing endonuclease 15 homolog	0.84	0.0266
RSRC2	arginine/serine-rich coiled-coil 2	0.84	0.0401
ZNF711	zinc finger protein 711	0.84	0.0072
TMEM106C	transmembrane protein 106C	0.84	0.0408
CHD7	chromodomain helicase DNA binding protein 7	0.83	0.0202
TPD52	tumor protein D52	0.83	0.0492
CD63	CD63 molecule	0.83	0.0102
KIAA1274	KIAA1274	0.83	0.0469
VASP	vasodilator-stimulated phosphoprotein	0.83	0.0473
CHD4	chromodomain helicase DNA binding protein 4	0.83	0.0382
CBX3	chromobox homolog 3	0.83	0.0299
IL13RA1	interleukin 13 receptor, alpha 1	0.83	0.0282
ELF2	E74-like factor 2	0.83	0.0363
HNRNPH3	heterogeneous nuclear ribonucleoprotein H3 (2H9)	0.83	0.0475
NMD3	NMD3 homolog	0.82	0.0183

CCDC47	coiled-coil domain containing 47	0.82	0.0366
VMP1	vacoule membrne protein 1	0.82	0.0312
RNF103	ring finger protein 103	0.82	0.0537
CST3	cystatin C	0.82	0.0274
RUNX1	runt-related transcription factor 1	0.82	0.0464
ARRDC3	arrestin domain containing 3	0.82	0.0497
NPM1	nucleophosmin 1	0.82	0.0484
S100A11P1	S100 calcium binding protein A11	0.82	0.0118
MAP3K7	mitogen-activated protein kinase kinase kinase 7	0.82	0.0398
BTBD10	BTB (POZ) domain containing 10	0.82	0.0077
CSRP3	cysteine and glycine-rich protein 3	0.82	0.0304
AZIN1	antizyme inhibitor 1	0.81	0.0126
TRIM5	tripartite motif-containing 5	0.81	0.0019
PNRC2	proline-rich nuclear receptor coactivator 2	0.81	0.0186
YWHAH	tyrosine 3-monooxygenase/tryptophan 5-monooxygenase activation protein eta	0.81	0.0377
PRKAG2	protein kinase, AMP-activated, gamma 2 non-catalytic subunit	0.81	0.0470
RBM25	RNA binding motif protein 25	0.81	0.0448
GPSM2	G-protein signaling modulator 2	0.81	0.0403
GALM	galactose mutarotase	0.81	0.0314
SEPHS2	selenophosphate synthetase 2	0.81	0.0084
PPP6R3	protein phosphatase 6 regulatory subunit 3	0.81	0.0355
TCEAL1	transcription elongation factor A (SII)-like 1	0.81	0.0318
LAMA4	laminin, alpha 4	0.81	0.0347
CNTRL	centriolin	0.81	0.0161
GNA13	guanine nucleotide binding protein alpha 13	0.81	0.0472
MAF	v-maf musculoaponeurotic fibrosarcoma oncogene homolog	0.81	0.0491
PNRC1	proline-rich nuclear receptor coactivator 1	0.81	0.0142
WNK1	WNK lysine deficient protein kinase 1	0.81	0.0541
GLT8D2	glycosyltransferase 8 domain containing 2	0.80	0.0522
WAPAL	wings apart-like homolog	0.80	0.0168
IER3IP1	immediate early response 3 interacting protein 1	0.80	0.0404
USP8	ubiquitin specific peptidase 8	0.80	0.0201
TUBA1A	tubulin, alpha 1a	0.80	0.0425
HFE	hemochromatosis	0.80	0.0300
MANBA	mannosidase, beta A, lysosomal	0.80	0.0413
CSDE1	cold shock domain containing E1, RNA-binding	0.80	0.0398
ACLY	ATP citrate lyase	0.80	0.0422
DERA	2-deoxyribose-5-phosphate aldolase homolog	0.80	0.0541
KDM3B	lysine (K)-specific demethylase 3B	0.80	0.0443
KDM1B	lysine demethylase 1B	0.80	0.0123
MAK16	MAK16 homolog	0.80	0.0532
C1orf21	chromosome 1 open reading frame 21	0.80	0.0345
TM9SF1	transmembrane 9 superfamily member 1	0.80	0.0405

DDX50	DEAD (Asp-Glu-Ala-Asp) box polypeptide 50	0.79	0.0343
CAMKK2	calcium/calmodulin-dependent protein kinase kinase 2, beta	0.79	0.0167
RARA	retinoic acid receptor, alpha	0.79	0.0153
PLRG1	pleiotropic regulator 1	0.79	0.0159
SNRNP40	small nuclear ribonucleoprotein 40kDa (U5)	0.79	0.0285
SRSF1	serine and arginine rich splicing factor 1	0.79	0.0237
RNF20	ring finger protein 20	0.78	0.0482
HNRNPH2	heterogeneous nuclear ribonucleoprotein H2	0.78	0.0187
PTGER3	prostaglandin E receptor 3	0.78	0.0374
LUC7L2	LUC7-like 2	0.78	0.0057
EXOSC2	exosome component 2	0.78	0.0295
ST6GAL2	ST6 beta-galactosamide alpha-2,6-sialyltranferase 2	0.78	0.0013
PLN	phospholamban	0.77	0.0192
RNF146	ring finger protein 146	0.77	0.0140
PDCD7	programmed cell death 7	0.77	0.0124
GPC4	glypican 4	0.77	0.0526
LGMN	legumain	0.77	0.0186
ABR	active BCR-related gene	0.76	0.0341
PRKAB2	protein kinase, AMP-activated, beta 2 non-catalytic subunit	0.76	0.0064
MBD4	methyl-CpG binding domain protein 4	0.75	0.0330
NUDT16	nudix hydroxylase 16	0.75	0.0186
TMEM87B	transmembrane protein 87B	0.75	0.0209
PALMD	palmdelphin	0.75	0.0375
CYFIP1	cytoplasmic FMR1 interacting protein 1	0.75	0.0287
THRAP3	thyroid hormone receptor associated protein 3	0.75	0.0399
FAM105A	family with sequence similarity 105, member A	0.74	0.0510
C9orf78	chromosome 9 open reading frame 78	0.74	0.0006
FCGRT	Fc fragment of IgG, receptor, transporter, alpha	0.74	0.0343
SPTBN2	spectrin, beta, non-erythrocytic 2	0.74	0.0361
FBXO21	F-box protein 21	0.74	0.0086
BAG2	BCL2-associated athanogene 2	0.74	0.0492
CALU	calumenin	0.73	0.0288
FABP5	fatty acid binding protein 5	0.73	0.0316
PDE4B	phosphodiesterase 4B, cAMP-specific	0.73	0.0196
SLMAP	sarcolemma associated protein	0.73	0.0438
CNOT10	CCR4-NOT transcription complex, subunit 10	0.73	0.0071
GATA6	GATA binding protein 6	0.73	0.0439
AKAP7	A kinase (PRKA) anchor protein 7	0.73	0.0256
SORBS2	sorbin and SH3 domain containing 2	0.73	0.0501
MITF	microphthalmia-associated transcription factor	0.73	0.0485
ENPP5	ectonucleotide pyrophosphatase/phosphodiesterase 5	0.70	0.0137
SAMD4A	sterile alpha motif domain containing 4A	0.70	0.0470
GLUL	glutamate-ammonia ligase	0.70	0.0045
TRIM45	tripartite motif-containing 45	0.70	0.0497

C20orf108	chromosome 20 open reading frame 108	0.68	0.0121
RASL11B	RAS-like, family 11, member B	0.68	0.0236
CSDA	cold shock domain protein A	0.67	0.0375
EEF1A1	eukaryotic translation elongation factor 1 alpha 1	0.67	0.0517
CAST	calpastatin	0.65	0.0144
TXNIP	thioredoxin interacting protein	0.64	0.0151
DNAJC21	DnaJ (Hsp40) homolog, subfamily C, member 21	0.64	0.0008
DPY19L1	dpy-19-like 1	0.63	0.0088
TMOD3	tropomodulin 3	0.62	0.0133
