# Supplementary material for: A Transcriptomic Model of Postnatal Cardiac Effects of Prenatal Maternal Cortisol Excess in Sheep
Source: Front Physiol. 2019 Jul 3;10:816. doi: 10.3389/fphys.2019.00816 (PMC6616147; doi:10.3389/fphys.2019.00816)
Supplement: Supplementary file 3 [file Table_3.pdf]

Supplemental Table 3: Differentially expressed genes ($p<0.05$) in Epicardial Adipose tissue of 2week old lambs after maternal cortisol treatment

Fold changes are expressed as values in lambs of cortisol-treated ewes compared to control lambs

**OFFICIAL SYMBOL**	**Gene Name**	**Fold Change**	**P.Value**
GBP2	guanylate binding protein 2, interferon-inducible	3.12	0.0093
FAM26F	family with sequence similarity 26, member F	1.87	0.0143
GSTM4	glutathione S-transferase mu 4	1.76	0.0074
GLRX	glutaredoxin (thioltransferase)	1.75	0.0219
SNHG12	small nucleolar RNA host gene 12 (non-protein coding)	1.72	0.0199
C10orf10	chromosome 10 open reading frame 10	1.70	0.0409
TOR3A	torsin family 3, member A	1.61	0.0085
TANC1	tetratricopeptide repeat, ankyrin repeat and coiled-coil containing 1	1.52	0.0033
FOS	v-fos FBJ murine osteosarcoma viral oncogene homolog	1.52	0.0143
IGH@	immunoglobulin heavy locus	1.51	0.0446
RGS3	regulator of G-protein signaling 3	1.50	0.0009
C16orf54	chromosome 16 open reading frame 54	1.46	0.0007
RGS1	regulator of G-protein signaling 1	1.46	0.0101
GBP7	guanylate binding protein 7	1.45	0.0369
GALP	galanin-like peptide	1.45	0.0114
TMCO1	transmembrane and coiled-coil domains 1	1.40	0.0244
DPT	dermatopontin	1.39	0.0161
ARNT	aryl hydrocarbon receptor nuclear translocator	1.38	0.0252
LOC615263		1.37	0.0371
PARG	similar to poly (ADP-ribose) glycohydrolase; poly (ADP-ribose) glycohydrolase	1.36	0.0281
CYP2G2P	cytochrome P450, family 2, subfamily G, polypeptide 2 pseudogene	1.36	0.0304
MAD2L2	MAD2 mitotic arrest deficient-like 2 (yeast)	1.35	0.0093
PRKG1	protein kinase, cGMP-dependent, type I	1.35	0.0192
HNRNPA2B1	heterogeneous nuclear ribonucleoprotein A2/B1	1.35	0.0128
NSRP1	nuclear speckle splicing regulatory protein 1	1.33	0.0209
PARVG	parvin, gamma	1.33	0.0442
DVL1	dishevelled, dsh homolog 1 (Drosophila)	1.33	0.0046
TRIB1	tribbles homolog 1 (Drosophila)	1.33	0.0353
FAM43A	family with sequence similarity 43, member A	1.31	0.0296
ITPKB	inositol 1,4,5-trisphosphate 3-kinase B	1.31	0.0473
LEP	leptin	1.31	0.0390
ANXA1	annexin A1	1.31	0.0391
KDR	kinase insert domain receptor	1.30	0.0348

COTL1	coactosin-like 1 (Dictyostelium)	1.30	0.0142
PFDN2	prefoldin subunit 2	1.29	0.0201
CLCN6	chloride channel 6	1.29	0.0156
GPR116	G protein-coupled receptor 116, name changed to adhesion related…	1.29	0.0025
ZFYVE26	zinc finger, FYVE domain containing 26	1.29	0.0226
WDR33	WD repeat domain 33	1.28	0.0481
EBF4	early B-cell factor 4	1.28	0.0413
FLT1	fms-related tyrosine kinase 1	1.28	0.0181
ANP32A	Acidic Nuclear Phosphoprotein 32 Family Member A	1.28	0.0321
PLOD1	procollagen-lysine 1, 2-oxoglutarate 5-dioxygenase 1	1.27	0.0313
DCAF7	WD repeat domain 68	1.27	0.0400
NOTCH4	Notch homolog 4 (Drosophila)	1.27	0.0449
ADCY4	adenylate cyclase 4	1.26	0.0171
TMCC1	transmembrane and coiled-coil domain family 1	1.26	0.0482
SAMSN1	SAM domain, SH3 domain and nuclear localization signals 1	1.26	0.0309
DAAM1	dishevelled associated activator of morphogenesis 1	1.25	0.0056
TNFRSF21	tumor necrosis factor receptor superfamily, member 21	1.25	0.0462
SETMAR	SET domain and mariner transposase fusion gene	1.25	0.0428
TAF5	TAF5 RNA polymerase II, TATA box binding protein (TBP)-associated factor, 100kDa	1.25	0.0060
MAP3K6	mitogen-activated protein kinase kinase kinase 6	1.25	0.0419
LURAP1L	leucine rich adaptor protein 1-like	1.24	0.0098
ZCCHC10	zinc finger, CCHC domain containing 10	1.24	0.0268
TEX10	testis expressed 10	1.24	0.0459
TASP1	taspase, threonine aspartase, 1	1.24	0.0478
C1orf85	chromosome 1 open reading frame 85	1.23	0.0421
PHACTR4	phosphatase and actin regulator 4	1.23	0.0302
RPS24	ribosomal protein S24	1.23	0.0321
MYOC	myocilin, trabecular meshwork inducible glucocorticoid response	1.23	0.0388
PELI1	pellino homolog 1 (Drosophila)	1.22	0.0429
CHRNE	cholinergic receptor, nicotinic, epsilon	1.22	0.0177
PCMT1	protein-L-isoaspartate (D-aspartate) O-methyltransferase	1.22	0.0403
PGAM1	phosphoglycerate mutase 1 (brain)	1.22	0.0413
ZNF90	zinc finger protein 90	1.21	0.0471
CDK19	cell division cycle 2-like 6 (CDK8-like)	1.21	0.0058
CTNND1	catenin (cadherin-associated protein), delta 1	1.21	0.0497
SUPT16H	suppressor of Ty 16 homolog (S. cerevisiae)	1.20	0.0279
PLEKHG4	pleckstrin homology domain containing, family G (with RhoGef domain) member 4	1.20	0.0129
ST6GALNAC2	ST6 (alpha-N-acetyl-neuraminyl-2,3-beta-galactosyl-1,3)-N-acetylgalactosaminide alpha-2,6-sialyltransferase 2	1.20	0.0487

NOTCH3	Notch homolog 3 (Drosophila)	1.20	0.0427
MMP14	matrix metallopeptidase 14 (membrane-inserted)	1.20	0.0364
FAM83H	family with sequence similarity 83, member H	1.20	0.0313
MEOX2	mesenchyme homeobox 2	1.19	0.0136
SEPT1	septin 1	1.19	0.0383
USHBP1	Usher syndrome 1C binding protein 1	1.19	0.0481
DYSF	dysferlin, limb girdle muscular dystrophy 2B (autosomal recessive)	1.19	0.0278
LMO2	LIM domain only 2 (rhombotin-like 1)	1.19	0.0468
RGAG4	retrotransposon gag domain containing 4	1.18	0.0331
PIAS2	protein inhibitor of activated STAT, 2	1.18	0.0270
UPF2	UPF2 regulator of nonsense transcripts homolog (yeast)	1.18	0.0402
KHNYN	KIAA0323	1.17	0.0412
KDM3A	lysine (K)-specific demethylase 3A	1.17	0.0391
DEK	DEK oncogene	1.17	0.0413
PCF11	PCF11, cleavage and polyadenylation factor subunit, homolog (S. cerevisiae)	1.17	0.0399
TMEM140	transmembrane protein 140	1.17	0.0427
STK19	serine/threonine kinase 19	1.16	0.0497
POLE3	polymerase (DNA directed), epsilon 3 (p17 subunit)	1.16	0.0423
C6orf132	chromosome 6 open reading frame 132	1.16	0.0249
IRF2BP2	interferon regulatory factor 2 binding protein 2	1.13	0.0407
APLP2	amyloid beta (A4) precursor-like protein 2	1.13	0.0393
BMP2K	BMP2 inducible kinase	1.13	0.0498
EP300	E1A binding protein p300	1.10	0.0411
ANAPC7	anaphase promoting complex subunit 7	0.89	0.0499
SGSM3	small G protein signaling modulator 3	0.89	0.0401
FKBP2	FK506 binding protein 2, 13kDa	0.89	0.0376
MAP3K7	mitogen-activated protein kinase kinase kinase 7	0.89	0.0192
USP14	ubiquitin specific peptidase 14 (tRNA-guanine transglycosylase)	0.88	0.0368
UBL3	ubiquitin-like 3	0.88	0.0410
VTI1B	vesicle transport through interaction with t-SNAREs homolog 1B (yeast)	0.88	0.0391
TTC1	tetratricopeptide repeat domain 1	0.88	0.0412
ERGIC2	ERGIC and golgi 2	0.88	0.0459
UBE2F	ubiquitin-conjugating enzyme E2F (putative)	0.87	0.0300
PIGP	phosphatidylinositol glycan anchor biosynthesis, class P	0.87	0.0424
GPANK1	G patch domain and ankyrin repeats 1	0.87	0.0226
RBM45	RNA binding motif protein 45	0.87	0.0293
TMEM186	transmembrane protein 186	0.87	0.0230
CHAC2	ChaC, cation transport regulator homolog 2 (E. coli)	0.87	0.0445
MFF	mitochondrial fission factor	0.87	0.0331

DNASE1L1	deoxyribonuclease I-like 1	0.87	0.0327
RAB10	RAB10, member RAS oncogene family	0.87	0.0083
CMBL	carboxymethylenebutenolidase homolog (Pseudomonas)	0.87	0.0454
WDR41	WD repeat domain 41	0.87	0.0177
CCT5	chaperonin containing TCP1, subunit 5 (epsilon)	0.87	0.0455
FXN	frataxin	0.87	0.0328
RWDD1	RWD domain containing 1-like 1; RWD domain containing 1	0.86	0.0374
COX5B	cytochrome c oxidase subunit Vb	0.86	0.0416
INTS10	integrator complex subunit 10	0.86	0.0237
RPLP2	ribosomal protein, large, P2 pseudogene 3	0.86	0.0474
LMBRD1	LMBR1 domain containing 1	0.86	0.0492
MRPL11	mitochondrial ribosomal protein L11	0.86	0.0281
DERL1	Der1-like domain family, member 1	0.86	0.0427
RPS25	ribosomal protein S25 pseudogene 8	0.86	0.0346
NHP2	NHP2 ribonucleoprotein homolog (yeast)	0.86	0.0291
HMGCS1	3-hydroxy-3-methylglutaryl-Coenzyme A synthase 1 (soluble)	0.86	0.0377
BTBD10	BTB (POZ) domain containing 10	0.85	0.0405
ASMTL	acetylserotonin O-methyltransferase-like	0.85	0.0489
SLC25A26	solute carrier family 25, member 26	0.85	0.0083
MTCH2	mitochondrial carrier homolog 2 (C. elegans)	0.85	0.0484
BCL2L13	BCL2-like 13 (apoptosis facilitator)	0.85	0.0137
DOHH	deoxyhypusine hydroxylase/monooxygenase	0.85	0.0110
PECR	peroxisomal trans-2-enoyl-CoA reductase	0.85	0.0360
NUBP1	nucleotide binding protein 1 (MinD homolog, E. coli)	0.85	0.0119
SEPW1	selenoprotein W, 1	0.85	0.0236
DTYMK	deoxythymidylate kinase (thymidylate kinase)	0.85	0.0402
WRB	tryptophan rich basic protein	0.85	0.0408
PAFAH1B2	platelet-activating factor acetylhydrolase, isoform Ib, subunit 2 (30kDa)	0.85	0.0446
NIPA2	non imprinted in Prader-Willi/Angelman syndrome 2	0.85	0.0149
MT-ND1	Mitochondrially Encoded NADH:Ubiquinone Oxidoreductase Core Subunit 1	0.85	0.0074
SLA2	Src-like-adaptor 2	0.85	0.0089
TM9SF1	transmembrane 9 superfamily member 1	0.85	0.0470
KIAA0020	KIAA0020	0.84	0.0417
MARCH6	membrane-associated ring finger (C3HC4) 6	0.84	0.0379
TBC1D7	TBC1 domain family, member 7	0.84	0.0143
AFF4	AF4/FMR2 family, member 4	0.84	0.0263
XRCC5	X-ray repair complementing defective repair in Chinese hamster cells 5	0.84	0.0477
UBE2G2	ubiquitin-conjugating enzyme E2G 2 (UBC7 homolog, yeast)	0.84	0.0129
ATPAF2	ATP synthase mitochondrial F1 complex assembly factor 2	0.84	0.0281

TMEM179B	transmembrane protein 179B	0.84	0.0228
HPCAL1	hippocalcin-like 1	0.84	0.0332
TMEM192	transmembrane protein 192	0.84	0.0246
IP6K1	inositol hexakisphosphate kinase 1	0.84	0.0343
GEMIN4	gem (nuclear organelle) associated protein 4	0.84	0.0071
CCDC56	coiled-coil domain containing 56	0.84	0.0179
MRPL43	mitochondrial ribosomal protein L43	0.83	0.0364
EXOSC2	exosome component 2	0.83	0.0052
MLYCD	malonyl-CoA decarboxylase	0.83	0.0439
TMEM134	transmembrane protein 134	0.83	0.0199
C9orf78	chromosome 9 open reading frame 78	0.83	0.0253
TRMT1	TRM1 tRNA methyltransferase 1 homolog (S. cerevisiae)	0.83	0.0425
TFAM	transcription factor A, mitochondrial	0.83	0.0200
RASGEF1A	RasGEF domain family, member 1A	0.83	0.0235
LAMP2	lysosomal-associated membrane protein 2	0.83	0.0318
GMDS	GDP-mannose 4,6-dehydratase	0.83	0.0273
ROBO2	roundabout, axon guidance receptor, homolog 2 (Drosophila)	0.83	0.0424
ESYT2	family with sequence similarity 62 (C2 domain containing), member B	0.83	0.0399
POC1B	WD repeat domain 51B	0.83	0.0438
TXN2	thioredoxin 2	0.83	0.0428
TCEB3B	transcription elongation factor B polypeptide 3B (elongin A2)	0.83	0.0070
WDR76	WD repeat domain 76	0.83	0.0472
OGN	osteoglycin	0.83	0.0232
SREK1IP1	SREK1-interacting protein 1	0.83	0.0160
B4GALT5	UDP-Gal:betaGlcNAc beta 1,4- galactosyltransferase, polypeptide 5	0.83	0.0037
TSEN15	tRNA splicing endonuclease 15 homolog (S. cerevisiae)	0.82	0.0186
CHURC1	churchill domain containing 1	0.82	0.0262
DCTPP1	dCTP pyrophosphatase 1	0.82	0.0266
RAP2A	RAP2A, member of RAS oncogene family	0.82	0.0339
TANGO2	transport and golgi organization 2 homolog (Drosophila)	0.82	0.0412
FAM204A	family with sequence similarity 204, member A	0.82	0.0220
HK1	hexokinase 1	0.82	0.0338
PSMB1	proteasome (prosome, macropain) subunit, beta type, 1	0.82	0.0191
C12orf24		0.82	0.0249
COX6B1	cytochrome c oxidase subunit Vib polypeptide 1 (ubiquitous)	0.82	0.0159
ARID1B	AT rich interactive domain 1B (SWI1-like)	0.81	0.0196
STK40	serine/threonine kinase 40	0.81	0.0342
COA7	cytochrome c oxidase assembly factor 7 (putative)	0.81	0.0393
WDR45	WD repeat domain 45	0.81	0.0335

ALDH5A1	aldehyde dehydrogenase 5 family, member A1	0.81	0.0218
EIF2S1	eukaryotic translation initiation factor 2, subunit 1 alpha, 35kD	0.81	0.0051
STK39	serine threonine kinase 39 (STE20/SPS1 homolog, yeast)	0.81	0.0054
GLRX5	glutaredoxin 5	0.80	0.0162
MIF4GD	MIF4G domain containing	0.80	0.0347
TM7SF2	transmembrane 7 superfamily member 2	0.80	0.0160
FAM185BP	family with sequence similarity 185, member B pseudogene	0.80	0.0123
VDAC1	voltage-dependent anion channel 1; similar to voltage-dependent anion channel 1	0.80	0.0185
PRADC1	protease-associated domain containing 1	0.80	0.0098
SFXN4	sideroflexin 4	0.80	0.0148
TSNAX	translin-associated factor X	0.80	0.0250
CNOT6	CCR4-NOT transcription complex, subunit 6	0.80	0.0225
C20orf29	chromosome 20 open reading frame 29	0.80	0.0123
NDUFAB1	NADH dehydrogenase (ubiquinone) 1, alpha/beta subcomplex, 1, 8kDa	0.79	0.0117
RNF146	ring finger protein 146	0.79	0.0231
C19orf48	chromosome 19 open reading frame 48	0.79	0.0420
PTGR2	prostaglandin reductase 2	0.79	0.0011
PRPF4	PRP4 pre-mRNA processing factor 4 homolog (yeast)	0.79	0.0035
COX6C	cytochrome c oxidase subunit VIc	0.79	0.0406
VPS13B	vacuolar protein sorting 13 homolog B (yeast)	0.79	0.0100
ECHS1	enoyl Coenzyme A hydratase, short chain, 1, mitochondrial	0.79	0.0171
TSN	translin	0.79	0.0088
TIPRL	TIP41, TOR signaling pathway regulator-like (S. cerevisiae)	0.79	0.0482
C11orf54	chromosome 11 open reading frame 54	0.79	0.0218
HSD17B1	hydroxysteroid (17-beta) dehydrogenase 1	0.78	0.0107
DCTN5	dynactin 5 (p25)	0.78	0.0147
MBD4	methyl-CpG binding domain protein 4	0.78	0.0495
ACACB	acetyl-Coenzyme A carboxylase beta	0.78	0.0449
HINT2	histidine triad nucleotide binding protein 2	0.78	0.0203
FHIT	fragile histidine triad gene	0.77	0.0185
MTFP1	mitochondrial fission process 1	0.77	0.0428
POLR1B	polymerase (RNA) I polypeptide B, 128kDa	0.77	0.0040
DLST	dihydrolipoamide S-succinyltransferase	0.77	0.0147
CRLS1	cardiolipin synthase 1	0.77	0.0376
FES	feline sarcoma oncogene	0.77	0.0029
MSI2	musashi homolog 2 (Drosophila)	0.77	0.0390
OBFC2A	oligonucleotide/oligosaccharide-binding fold containing 2A	0.77	0.0021
ALDH1A1	aldehyde dehydrogenase 1 family, member A1	0.77	0.0040
TNFRSF13B	tumor necrosis factor receptor superfamily, member 13B	0.76	0.0251

HADHB	hydroxyacyl-Coenzyme A dehydrogenase/3-ketoacyl-Coenzyme A thiolase/enoyl-Coenzyme A hydratase (trifunctional protein), beta subunit	0.76	0.0279
TMEM106C	transmembrane protein 106C	0.76	0.0354
UCK1	uridine-cytidine kinase 1	0.76	0.0125
MRPL46	mitochondrial ribosomal protein L46	0.75	0.0182
COX16	COX16 cytochrome c oxidase assembly homolog (S. cerevisiae)	0.75	0.0181
DLD	dihydrolipoamide dehydrogenase	0.75	0.0052
EPHX2	epoxide hydrolase 2, cytoplasmic	0.75	0.0030
PANK1	pantothenate kinase 1	0.75	0.0354
HMGCS2	3-hydroxy-3-methylglutaryl-Coenzyme A synthase 2 (mitochondrial)	0.73	0.0481
INSIG1	insulin induced gene 1	0.73	0.0302
KCNE3	potassium voltage-gated channel, Isk-related family, member 3	0.72	0.0147
ACSF2	acyl-CoA synthetase family member 2	0.72	0.0253
TXNDC9	thioredoxin domain containing 9	0.72	0.0271
RBM47	RNA binding motif protein 47	0.72	0.0397
PCDHB16	protocadherin beta 16	0.71	0.0329
PON3	paraoxonase 3	0.71	0.0138
TRO	trophinin	0.71	0.0107
SOD2	superoxide dismutase 2, mitochondrial	0.71	0.0016
BIK	BCL2-interacting killer (apoptosis-inducing)	0.71	0.0252
SBSN	suprabasin	0.71	0.0361
FANCD2	Fanconi anemia, complementation group D2	0.70	0.0128
ARPC3	actin related protein 2/3 complex, subunit 3	0.70	0.0137
DNAJC21	DnaJ (Hsp40) homolog, subfamily C, member 21	0.70	0.0064
F3	coagulation factor III (thromboplastin, tissue factor)	0.70	0.0322
ESR2	estrogen receptor 2 (ER beta)	0.69	0.0446
ADA	adenosine deaminase	0.69	0.0380
TNMD	tenomodulin	0.68	0.0275
C4orf47	chromosome 4 open reading frame 47	0.67	0.0275
NUDT15	nudix (nucleoside diphosphate linked moiety X)-type motif 15	0.66	0.0185
PEX12	peroxisomal biogenesis factor 12	0.66	0.0112
TMEM87B	transmembrane protein 87B	0.65	0.0302
SOD1	superoxide dismutase 1, soluble	0.65	0.0029
NPPC	natriuretic peptide precursor C	0.63	0.0078
CA2	carbonic anhydrase II	0.63	0.0409
CAB39L	calcium binding protein 39-like	0.61	0.0004
DHRS13	dehydrogenase/reductase (SDR family) member 13	0.61	0.0476
PLIN2	adipose differentiation-related protein	0.60	0.0482
GK	glycerol kinase 3 pseudogene; glycerol kinase	0.57	0.0223

UGT1A7	UDP glucuronosyltransferase 1 family, polypeptide A7	0.55	0.0075
FBP2	fructose-1,6-bisphosphatase 2	0.54	0.0186
MID1IP1	MID1 interacting protein 1 (gastrulation specific G12 homolog (zebrafish))	0.54	0.0084
ACLY	ATP citrate lyase	0.53	0.0133
LTF	lactotransferrin	0.47	0.0002
